# Analysis of 3.5 million SARS-CoV-2 sequences reveals unique mutational trends with consistent nucleotide and codon frequencies

**DOI:** 10.1186/s12985-023-01982-8

**Published:** 2023-02-17

**Authors:** Sarah E. Fumagalli, Nigam H. Padhiar, Douglas Meyer, Upendra Katneni, Haim Bar, Michael DiCuccio, Anton A. Komar, Chava Kimchi-Sarfaty

**Affiliations:** 1 Hemostasis Branch, Division of Plasma Protein Therapeutics, Office of Tissues and Advanced Therapies, Center for Biologics Evaluation and Research, US Food and Drug Administration, Silver Spring, MD USA; 2 Department of Statistics, University of Connecticut, Storrs, CT USA; 3 Present Address: Rockville, USA; 4 Department of Biological, Geological and Environmental Sciences, Center for Gene Regulation in Health and Disease, Cleveland State University, Cleveland, OH USA

**Keywords:** SARS-CoV-2, Nucleotide usage, Codon usage bias, Relative synonymous codon usage, Codon adaptation index and dN/dS

## Abstract

**Background:**

Since the onset of the SARS-CoV-2 pandemic, bioinformatic analyses have been performed to understand the nucleotide and synonymous codon usage features and mutational patterns of the virus. However, comparatively few have attempted to perform such analyses on a considerably large cohort of viral genomes while organizing the plethora of available sequence data for a month-by-month analysis to observe changes over time. Here, we aimed to perform sequence composition and mutation analysis of SARS-CoV-2, separating sequences by gene, clade, and timepoints, and contrast the mutational profile of SARS-CoV-2 to other comparable RNA viruses.

**Methods:**

Using a cleaned, filtered, and pre-aligned dataset of over 3.5 million sequences downloaded from the GISAID database, we computed nucleotide and codon usage statistics, including calculation of relative synonymous codon usage values. We then calculated codon adaptation index (CAI) changes and a nonsynonymous/synonymous mutation ratio (dN/dS) over time for our dataset. Finally, we compiled information on the types of mutations occurring for SARS-CoV-2 and other comparable RNA viruses, and generated heatmaps showing codon and nucleotide composition at high entropy positions along the Spike sequence.

**Results:**

We show that nucleotide and codon usage metrics remain relatively consistent over the 32-month span, though there are significant differences between clades within each gene at various timepoints. CAI and dN/dS values vary substantially between different timepoints and different genes, with Spike gene on average showing both the highest CAI and dN/dS values. Mutational analysis showed that SARS-CoV-2 Spike has a higher proportion of nonsynonymous mutations than analogous genes in other RNA viruses, with nonsynonymous mutations outnumbering synonymous ones by up to 20:1. However, at several specific positions, synonymous mutations were overwhelmingly predominant.

**Conclusions:**

Our multifaceted analysis covering both the composition and mutation signature of SARS-CoV-2 gives valuable insight into the nucleotide frequency and codon usage heterogeneity of SARS-CoV-2 over time, and its unique mutational profile compared to other RNA viruses.

**Supplementary Information:**

The online version contains supplementary material available at 10.1186/s12985-023-01982-8.

## Background

Since the emergence in late 2019, the novel severe acute respiratory syndrome coronavirus 2 (SARS-CoV-2) has spread globally and caused the coronavirus disease 2019 (COVID-19) pandemic with very serious medical sequelae for many individuals [1, 2]. Rapid development and introduction of vaccines against SARS-CoV-2 within a year, by late 2020, has contributed to significantly lowering the transmission, severity of illness and mortality from COVID-19 [3]. Despite this unprecedented effort, SARS-CoV-2 continued to mutate and evolve resulting in the emergence of new strains and resulted in multiple waves of new infections across the globe [4]. Specifically, the World Health Organization (WHO) designated 5 strains—Alpha, Beta, Gamma, Delta, and Omicron as variants of concern (VOC) due to either increase in transmission, virulence, or escape from existing diagnostics, vaccine immunity, and therapeutics [5–7]. The continued emergence of SARS-CoV-2 strains with higher fitness suggests the need for continued monitoring of viral evolution and identification of features contributing to viral fitness.

Evaluation of nucleotide composition and synonymous codon usage bias of viral genomes is useful to understand viral genetic changes, immune evasion and its adaptation to host [8]. Degeneracy of genetic code, referring to the availability of 64 possible codons to code for 20 amino acids, allows a majority of amino acids to be encoded by more than one codon. These synonymous codons of an amino acid are not used uniformly and usage of some codons is preferred over others, a phenomenon known as codon usage bias (CUB) [9]. CUB is primarily shaped by mutational and translational pressures which correlate with multiple factors including translation efficiency/fidelity (abundance of tRNAs), gene expression, location within genes, nucleotide composition, and mRNA/protein structure [10, 11]. Relative synonymous codon usage (RSCU) [12] and codon adaptation index (CAI) [13] are a couple of metrics commonly used to measure CUB [14, 15]. Scaled ratio of nonsynonymous to synonymous variants, referred to as dN/dS, is often calculated in order to understand the direction and magnitude of selection at the molecular level [16]. However, interpretation of results from such analysis assumes that synonymous variants are neutral. A growing body of literature in the last 2 decades demonstrated that synonymous variants could affect expression and quality attributes of the encoded protein by multiple mechanisms at both transcriptional and translational levels through their effects on pre-mRNA splicing, mRNA structure/stability, miRNA binding, translation efficiency, and co-translational folding [17]. Additionally, RNA structures play an important role in the translation and replication of RNA viruses, including SARS-CoV-2, and a selection pressure against synonymous variants in these functional regions was reported [18]. Therefore, synonymous variants cannot be assumed neutral and careful interpretations of results is needed [19].

SARS-CoV-2 has a positive sense single strand RNA genome of ~ 30 kb in length [20, 21]. About 2/3 of the genome at 5′ end comprises of two overlapping reading frames ORF1a and ORF1ab. The distal 1/3 of the genome encodes for 4 structural proteins: Spike (S), Envelope (E), Membrane (M), Nucleocapsid (N), and additional accessory proteins (ORF-3a, -3b, -6, -7a, -7b, -8, and -10). The Spike protein is a surface glycoprotein that is essential for viral attachment and entry in to host cells. M and E proteins are also located on the surface of virion with functional roles in viral assembly and pathogenesis. The N protein is located within the viral capsid and is involved in viral RNA packaging. Similar to other coronaviruses, SARS-CoV-2 has an AT rich genome (~ 62% AT and 38% GC content). T nucleotide is most used (~ 34%) followed by A (~ 28%), C (~ 20%), and G (18%) nucleotides. Subsequently, RSCU analysis showed that a majority of preferentially used codons in the SARS-CoV-2 genome are T or A ending while G and C ending codons predominated the less preferred ones [22]. Over a period of time, a significant increase in T usage with simultaneous decrease in C usage, primarily through C—> T transitions mediated by the action of host cell APOBEC cytosine deaminases was reported [23]. A minimal, albeit significant decrease in the CAI values of SARS-CoV-2 genomes over time was reported. This decrease over time was reported to be a result of lower CAI values of later emergent strains like Alpha and Delta rather than decrease within strains. Interestingly, the latest VOC, Omicron was reported to have CAI higher than other VOC, but lower than the original Wuhan-Hu-1 strain [24].

Previous studies analyzed a limited number of sequences and strains over a short period of time. In this study, we sought to perform a variety of bioinformatic analyses on the largest possible cohort of SARS-CoV-2 sequences for the entire timespan of the COVID-19 pandemic. We analyzed 3,573,491 sequences downloaded from GISAID, a substantially larger cohort than comparable analyses [24–27], by measuring nucleotide and codon frequencies, RSCU, and CAI over time separated by month and clade. Additionally, we analyzed dN/dS ratios for individual clades of SARS-CoV-2 and visualized the frequencies of synonymous and nonsynonymous mutations for Spike gene of SARS-CoV-2 Omicron and Delta clades, and analogous genes in Middle East Respiratory Syndrome Coronavirus (MERS-CoV), Influenza, and Dengue viruses. Altogether, these analyses showed changes in nucleotide and codon usage characteristics of SARS-CoV-2 genome over time and revealed its unique mutation pattern compared to some other RNA viruses.

## Methods

### Design of study

**Fig. 1:**
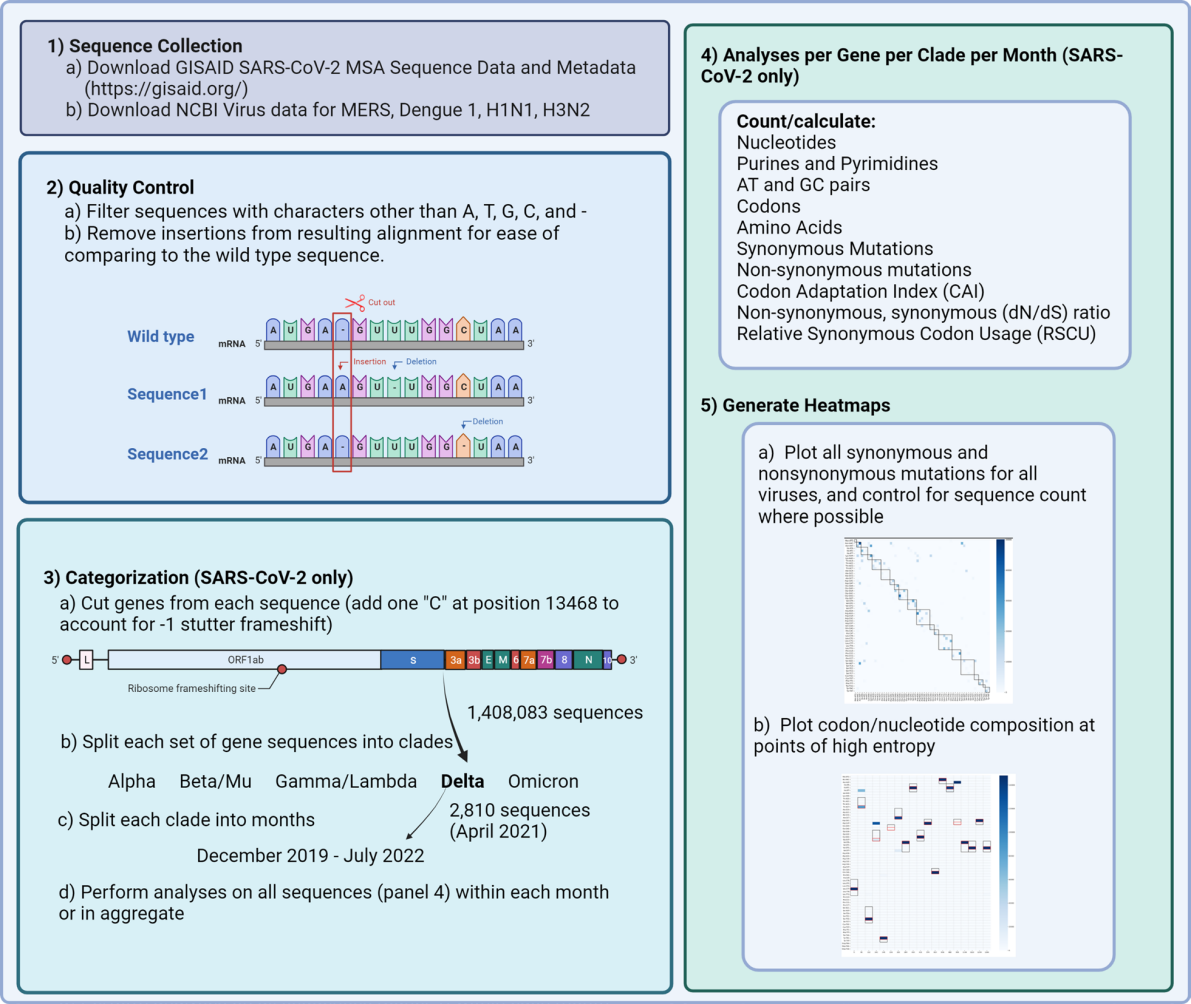
Bioinformatics Workflow Diagram Showing Sequence of Data Collection, Processing, and Presentation

We started by calculating nucleotide and codon usage frequencies for all sequences, separated by gene, month, and clade. We then calculated RSCU over time with specific focus on codons that either had specific requirements (specialized translation factor, eIF5A) for translation/decoding (proline) or had particularly distinct RSCU values. We expanded these calculations to include CAI and dN/dS ratio, which respectively gave insight into viral similarity to the host and whether nonsynonymous mutations were favored. Finally, we visualized the synonymous and nonsynonymous mutations for SARS-CoV-2 with specially designed heatmaps, focusing on Omicron and Delta clades, and compared with those generated for Influenza, MERS-CoV, and Dengue sequences. Our approach is summarized in Fig. 1.

### Data collection

The complete reference sequence for SARS-CoV-2 (GISAID accession EPI_ISL_402124; NCBI accession NC_045512.2), referred to as wild-type (WT) from here onwards and multiple sequence alignments (MSA) for about 10 million complete genome sequences of SARS-CoV-2 isolates were downloaded from the GISAID SARS-CoV-2 datahub (accessed on August 21, 2022). We also downloaded the GISAID metadata file for finer details associated with each sample [28]. The reader can also interact with some of the metadata on the GISAID sister website CoVariants (https://covariants.org). This resource provides a breakdown of some of GISAID SARS-CoV-2 metadata, following each variant and some lineages over time via individual non-synonymous and synonymous mutations for each gene. This site also provides per country change in the frequencies of each variant, an estimated cases tracker, protein visualization, and a table of shared variant mutations. The data we downloaded contains no duplicates or low-quality sequences (> 5% undefined bases); and we further filtered out sequences that contained ambiguous characters other than A, T, G, and C.

The open reading frame (ORF) sequences of 12 genes (ORF1ab, S, ORF3a, ORF3b, E, M, ORF6, ORF7a, ORF7b, ORF8, N, and ORF10) were trimmed from each genomic sequence according to GISAID nucleotide locations and analyzed independently (the 12 genes do not overlap). For ease of comparison between the WT and subsequent gene sequences, any insertion found in the WT sequence was removed from all sequences (Fig. 1). Deletions found in subsequent sequences were kept. MSA gene sequences with minimal modification were used for counting synonymous and nonsynonymous mutations, and for comparison to the number of mutations in other viruses. This modification introduces a duplication of the shared C nucleotide between nsp11 and nsp12 (frameshift of ORF1ab, nucleotide location—13,203) and applied to all SARS-CoV-2 sequences.

The MSA gene sequence data were split into clades as assigned by GISAID (Alpha (GRY), Beta/Mu (GH), Gamma/Lambda (GR), Delta (GK), and Omicron (GRA)), and then by collection date (by month spanning December 2019–July 2022). Collection date was chosen over submission date (often several months apart) to better represent when a particular variant sequence existed. GISAID derived their clade nomenclature based on phylogenetic groupings [29]. Table 1 summarizes the resulting 3,573,491 filtered MSA sequences (from herein simply called SARS-CoV-2 sequences) broken down by clade and month. Of note, the chronology of the GISAID clades with their associated WHO labels (Alpha, Beta/Mu, Omicron, etc.) do not line up perfectly with the chronology of the virus as outlined by the WHO—for example, GISAID grouped multiple sequences labeled “GRA/Omicron” as early as December 2019, even though Omicron probably did not emerge until December 2021 per the WHO. To account for this, within Table 1 we highlighted in bold italic font the months for each clade that were both: (1) after the earliest detection date for that clade as defined by the WHO, and (2) had an adequate number of sequences, arbitrarily set at 5000. We did this in order to rule out months which may have misclassified sequences, or which did not have enough sequences to provide meaningful data. The highlighted months were the focus of most of our analyses.

**Table 1:** Sequence Counts by Month and Clade

Month	Alpha	Beta/Mu	Gamma/Lambda	Delta	Omicron	Month Total
19-Dec	2	9	5	2	5	23
20-Jan	77	162	111	33	53	436
20-Feb	56	246	210	71	77	660
20-Mar	155	9526	4705	178	260	14,824
20-Apr	52	8377	***7797***	58	54	16,338
20-May	17	***5085***	***4748***	28	12	9890
20-Jun	16	***5075***	***7371***	7	16	12,485
20-Jul	7	***6362***	***12,014***	5	9	18,397
20-Aug	6	***5480***	***11,240***	16	12	16,754
20-Sep	16	***6824***	***8140***	20	16	15,016
20-Oct	65	***8703***	***8449***	20	4	17,241
20-Nov	1426	***14,171***	***10,442***	6	–	26,045
20-Dec	***10,903***	***21,645***	***12,549***	27	15	45,139
21-Jan	***36,946***	***30,750***	***18,350***	117	32	86,195
21-Feb	***64,847***	***24,422***	***15,173***	93	18	104,553
21-Mar	***130,982***	***24,750***	***23,150***	597	14	179,493
21-Apr	***132,923***	***18,068***	***32,294***	2810	27	186,122
21-May	***91,528***	***8342***	***33,667***	***17,588***	18	151,143
21-Jun	**23,524**	3881	***24,279***	***47,694***	50	99,428
21-Jul	2955	2747	***19,767***	***170,492***	44	196,005
21-Aug	363	1074	***8543***	***303,252***	16	313,248
21-Sep	36	631	1187	***239,152***	9	241,015
21-Oct	11	397	220	***197,488***	7	198,123
21-Nov	6	510	147	***248,064***	296	249,023
21-Dec	14	480	143	***166,615***	***63,644***	230,896
22-Jan	2	32	20	***12,964***	***291,817***	304,835
22-Feb	3	5	11	550	***253,746***	254,315
22-Mar	–	2	2	98	***233,137***	233,239
22-Apr	–	2	1	25	***132,539***	132,567
22-May	–	–	6	5	***133,097***	133,108
22-Jun	–	–	–	8	***85,050***	85,058
22-Jul	–	–	–	–	1877	1877
Clade Total	496,938	207,758	264,741	1,408,083	1,195,971	3,573,491

The numbers in bold italic represent those months which both (1) are after the date that the clade was first noted by the WHO and (2) contain over 5,000 sequences

We obtained sequences of other RNA viruses to perform mutation comparisons, selected based on the number of available sequences and by the presence of an analogous protein to SARS-CoV-2 spike. Sequences of H1N1 (NCBI Accession NC_026433.1) and H3N2 (NCBI Accession NC_007366.1) strains of Influenza, Dengue 1 (NCBI Accession NP_722460.2) strain of Dengue virus, and MERS-CoV (NCBI Accession NC_019843) sequences were all downloaded from NCBI virus database (accessed on September 15, 2022). Filters were applied to eliminate sequences containing ambiguous characters and those obtained from non-human hosts. Only coding sequences for proteins that were functionally similar to SARS-CoV-2 Spike were downloaded to facilitate ease of comparison. These include Hemagglutinin (HA) for H1N1 and H3N2, Envelope (E) for Dengue 1, and Spike for MERS-CoV. HA has been shown to have antigenic sites and is primarily responsible for entry into the host cell [30]. E in Dengue is responsible for both virion assembly and merging the virus with host cells [31]. Spike in MERS-CoV is highly similar and closely related in function to Spike in SARS-CoV-2, enabling the virus to bind to cellular receptors and mediating cell entry [32].

### Nucleotide and codon usage analysis

For each of the five clades across the 32 months, the nucleotide (A, T, C, G), purine (AG), pyrimidine (TC), adenine–thymine (AT), and guanine-cytosine (GC) frequencies were calculated for each of the 12 SARS-CoV-2 genes (listed earlier) independently. Gene sequences were then parsed into codons, and the codon and corresponding amino acid (AA) frequencies were calculated for all clades at all time points.

### Relative synonymous codon usage (RSCU) analysis

To assess CUB, RSCU was calculated as described previously [15, 33] for all clades across 32 months. An RSCU value of 1 indicates lack of CUB for the specific codon. If codon usage is biased, the RSCU for at least one synonymous codon of an amino acid will skew from one. We arbitrarily considered any RSCU value over 1.5 as ‘highly overrepresented’ and an RSCU value under 0.5 as ‘highly underrepresented’ [34, 35]. We only calculated RSCU for genes with > 1000 nucleotides (ORF1ab, Spike, and N gene) as it is less likely a codon is represented just by chance.

### Codon adaptation index (CAI) analysis

We calculated CAI for the months highlighted in bold italic font in Table 1 using a comparable approach to that described previously [36, 37]. CAI values range from 0 to 1 with a value of 1 indicating most bias to the reference (in this study, the reference is *Homo sapiens)*.

### dN/dS calculations

We calculated the dN/dS ratio as it changed over time for only the months highlighted in bold italic font in Table 1. dN/dS is well established in the literature as a metric applied to protein coding genes [38]. dN/dS is the ratio of nonsynonymous and synonymous mutations, adjusted for the number of nonsynonymous and synonymous sites. A value greater than 1 indicates that nonsynonymous changes are favored by selection, while a value less than 1 indicates that nonsynonymous changes are disfavored. We tabulated missense and synonymous mutations for all the viruses analyzed.

### Statistical analysis

Significance was calculated between clades at each time point for a particular trait of a gene. For example, we tested whether Alpha’s Spike gene Adenine nucleotide distribution is significantly different than Beta/Mu’s Spike gene Adenine nucleotide distribution during January 2021. These comparisons were calculated per gene per month for each nucleotide and codon. We used Python’s (version 3.8) SciPy library [39] and Pandas [40] to run a Welch’s t-test and find the raw *p*-values for each of the tests performed. *p*-values were adjusted using the Bonferroni correction (0.05/N), where significance is dependent on the number of tests performed (N). If the raw *p*-value is less than the adjusted threshold, the null hypothesis is rejected.

### Figure preparation

All figures were created using Matplotlib 3.5.1. [41], BioRender, and Graphpad Prism 9.4.1.

## Results

In the current study, 3,573,491 SARS-CoV-2 genome sequences downloaded from the GISAID website and assigned to Alpha, Beta/Mu, Gamma/Lambda, Delta, and Omicron clades were analyzed (Table 1).

### Nucleotide frequency over time

**Fig. 2:**
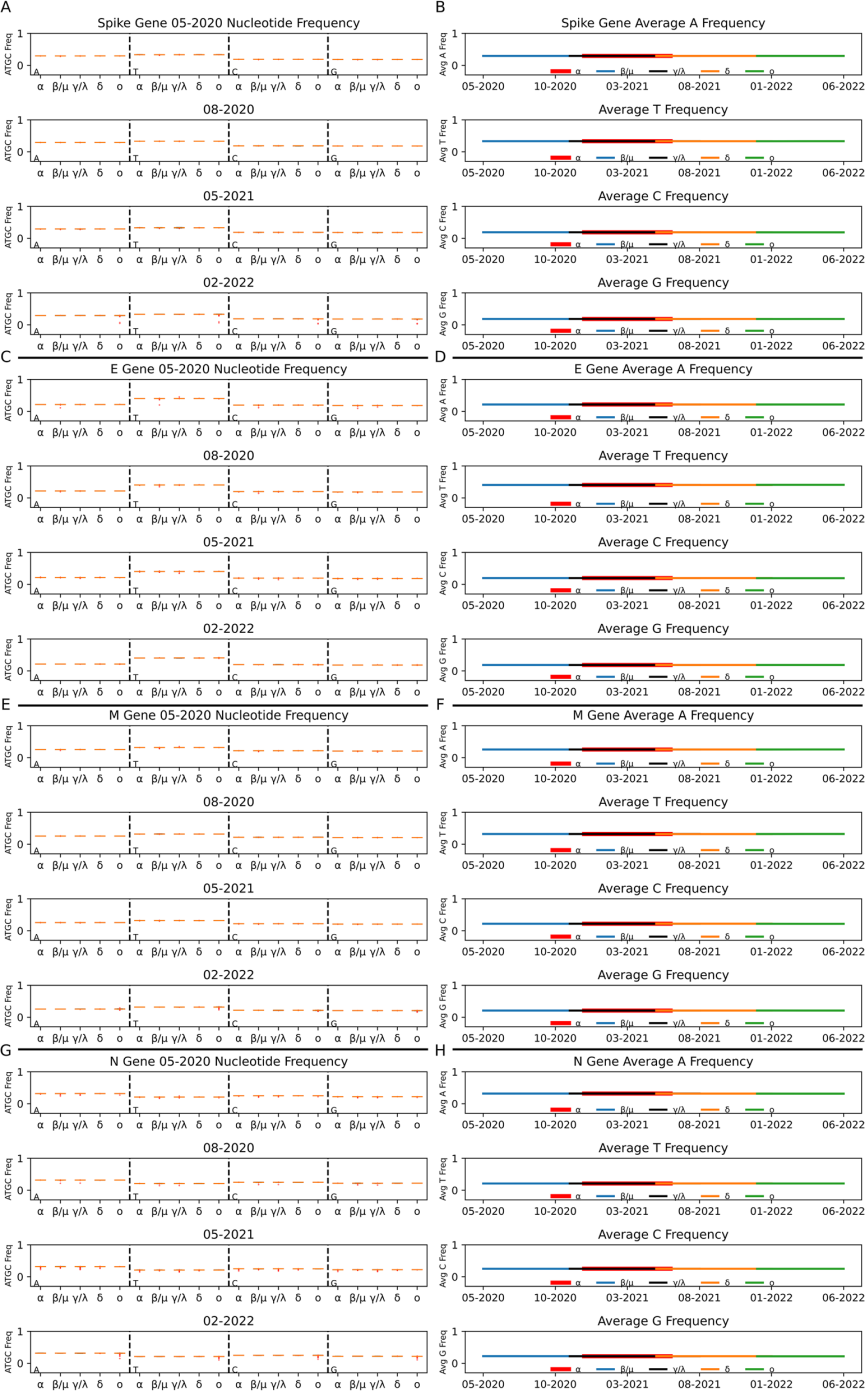
Minimal Differences in Nucleotide Frequency Between Clades, Over Time Across Structural Genes. Clades are represented by their Greek letter and corresponding color for line graphs: Alpha—α (red), Beta/Mu—β/μ (blue), Gamma/Lambda—γ/λ (black), Delta—δ (orange), and Omicron—ο (green). Within each box plot, orange bars represent the median nucleotide frequency of each clade distribution, red dots represent outliers, and black boxes represent the first and third quartiles (often hidden behind the orange median bar). **A** Spike gene nucleotide frequency distributions over select months. **B** Spike gene average nucleotide frequencies plotted over significantly large sampling months. **C** E gene nucleotide frequency distributions over select months. **D** E gene average nucleotide frequencies plotted over significantly large sampling months. **E** M gene nucleotide frequency distributions over select months. **F** M gene average nucleotide frequencies plotted over significantly large sampling months. **G** N gene nucleotide frequency distributions over select months. **F** N gene average nucleotide frequencies plotted over significantly large sampling months. All line graphs utilized the bold regions of the Table 1 clade distributions

To quantify the nucleotide usage variation within and between clades, we calculated the frequency of nucleotides both individually (A, T, C, G, appear in this order for each month/clade) and grouped as purines (sum of A and G), pyrimidines (sum of T and C), adenine–thymine (sum of A and T), and guanine-cytosine (sum of G and C) over the period of 32 months for individual genes: ORF1ab, Spike, ORF3a, ORF3b, E, M, ORF6, ORF7a, ORF7b, ORF8, N, and ORF10. Despite the presence of mutations defining the clades themselves, our analysis revealed very low variation within and between the clades across 32 months for all genes. This low variation is demonstrated by the observed nucleotide frequency distributions over select months for the structural genes (Spike, E, M, and N) from all 5 clades in Fig. 2. Corresponding graphs and data for all other genes (ORF1ab, ORF3a, ORF3b, ORF6, ORF7a, ORF7b, ORF8, and ORF10) can be found in Additional files. For most distributions in Fig. 2, all samples of a clade are very close in frequency to the median (orange bar), making the interquartile range (black box) and whiskers (black vertical lines) indistinct. Based on these data, nucleotide usage frequency for most gene sequences is observed as T > A > C > G. Specifically, Spike, E, and M genes with high usage of T nucleotide showed similar usage frequencies of A, T, C and G nucleotides to most genes within and between all clades for the select months of May 2020, October 2020, May 2021 and February 2022 (Fig. 2A, C, E, and G, respectively—these months were chosen because they each contain samples from each clade) (variance data can be found in Additional file 1: Table S1). Interestingly, the nucleotide usage of the N gene is unique in comparison to other structural genes and is consistently biased towards A nucleotides rather than T (Fig. 2G: A > C > G > T). Analysis of nucleotide frequencies for the Spike, E, M, and N genes over the largest sampling months per clade (highlighted by bold italic font in Table 1) also showed largely similar usage of nucleotides (Fig. 2B, D, F, H, respectively).

Despite the apparent low variation in the structural gene’s nucleotide usage, statistical analysis of clade distributions within each month revealed many significant differences (Additional file 13: Figure S1). All comparisons were calculated using a Welch’s t-test, assuming variance is unequal and applying the Bonferroni correction (adjusted alpha). Statistical results are summarized in Table 2 and 3 and all the data can be found in Additional file 2: Table S2 and Additional file 3: Table S3. The greatest number of significant differences between clade nucleotide distributions for Spike, E, M, and N genes all resulted from different months, though similar in number (Table 2). When these differences are summed across clades and months, the number of significant differences between nucleotides varies widely for the Spike, E, M, and N genes (Table 3). The differences seen across nucleotide frequencies and months is to be expected with random mutations.

**Table 2:** SARS-CoV-2 Structural Gene Nucleotide Significant Difference Analysis Highlighting Specific Months

Gene	*Spike*	*E*	*M*	*N*
Month with greatest number of significant differences (listed -summed across all clades and nucleotides)	July 2021: 38 differences	December 2020: 33 differences	December 2021: 28 differences	April 2021: 38 differences
Range of significant *p*-values	1.94E^−05^–0	0.001–9.75E^−116^	0.001–0	0.001–0
Adjusted alpha	0.0125	0.0014	0.0013	0.0125

Data from which this table was created can be found in Additional file 2: Table S2 and Additional file 3: Table S3

**Table 3:** Total SARS-CoV-2 Nucleotide Significant Differences Across All Clades and Timepoints

Gene →	*Spike*	*E*	*M*	*N*
Nucleotide ↓				
A	140	55	98	172
T	103	109	140	116
C	116	111	136	147
G	145	109	105	156

Data from which this table was created can be found in Additional file 2: Table S2 and Additional file 3: Table S3

This low variation trend continues for the structural genes regarding adenine–thymine/ guanine-cytosine (AT/GC) and purine/pyrimidine (AG/TC) frequency distributions (Additional file 14: Figure S2 and Additional file 15: Figure S3 respectively, and Additional file 4: Table S4 and Additional file 5: Table S5). We also analyzed nucleotide, purine/pyrimidine (AG/TC) and adenine–thymine/guanine-cytosine (AT/GC) frequency data for ORF1ab, ORF3a, ORF3b, ORF6, ORF7a, ORF7b, ORF8, and ORF10 (Additional file 5: Table S5, Additional file 6: Table S6, Additional file 16: Figure S4, Additional file 17: Figure S5, Additional file 18: Figure S6, Additional file 19: Figure S7, Additional file 20: Figure S8, and Additional file 21: Figure S9). These non-structural genes also express very little variation within and between clade distributions over time. Of note, ORF6 gene nucleotide usage data is biased towards the A nucleotide (A > T > C > G), and ORF8 gene uses the G nucleotide slightly more often than C (T > A > G > C). Also, genes ORF7a, ORF8, and ORF10 contain many outliers in comparison to other genes (Additional file 21: Figure S9).

Very minimal nucleotide variation is present within and between clades for each gene when the y-axis is scaled between 0 and 1. The variance in nucleotide frequency fluctuates over time much more so than the average of these clade distributions for each gene (Additional file 1: Table S1 and Additional file 6: Table S6). Also, the average and variance of these distributions over time for all clades are shown to positively covary for Spike (4.73E^−11^), ORF3a (1.36E^−08^), ORF3b (1.89E^−08^), E (2.51E^−08^), ORF6 (2.74E^−08^), ORF7a (2.28E^−07^), N (7.34E^−09^), and ORF10 (3.34E^−06^), and negatively covary for ORF1ab (− 2.13E^−10^), M (− 3.89E^−10^), ORF7b (− 6.60E^−07^), and ORF8 (− 3.93E^−09^) genes (Additional file 1: Table S1 and Additional file 6: Table S6). Interestingly, the nucleotide variance over time for Omicron tends to fluctuate away from Alpha, Beta/Mu, Gamma/Lambda, and Delta, which are often the same. However, there is an average increase in nucleotide variance over time for most genes/clades, peaking in late 2021 and into spring 2022 (Additional file 1: Table S1).

### Codon usage bias over time

Following the observation of very low variation between and within clades at the nucleotide level, we performed variation analysis on the level of codons to better understand the usage of specific synonymous codons unique to each clade. Our codon analysis across all genes revealed very minimal variation between and within clades over the 32-month sampling period. Statistical analysis between clades per month were conducted as described for the nucleotide data above.

**Fig. 3:**
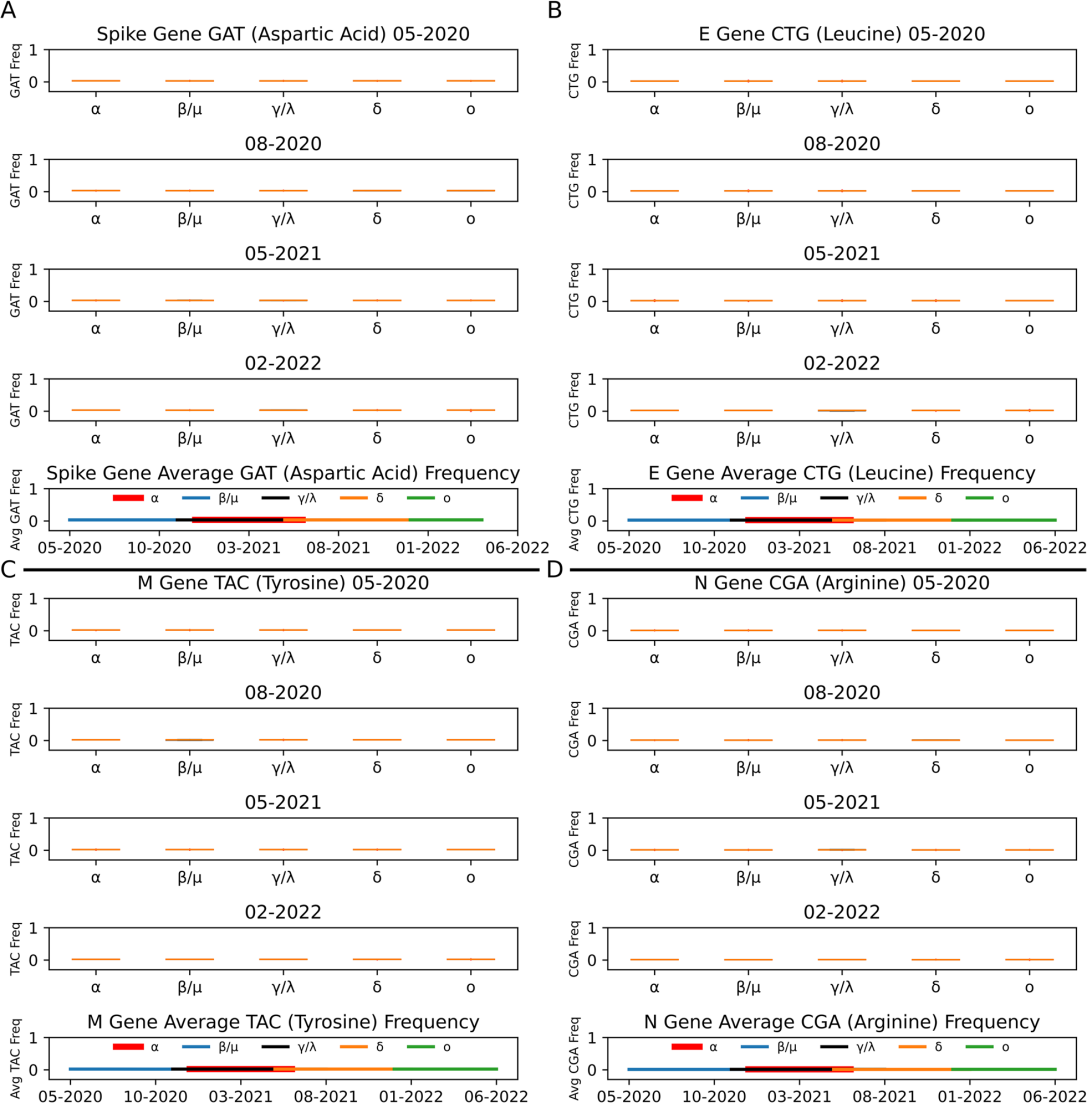
Codon Usage of Structural Genes Remains Stable Between Clades and Over Time. Codons highlighted here were selected based on greatest number of significant differences between December 2019 and July 2022 (Additional file 7: Table S7). Clades are represented by their Greek letter and corresponding color for line graphs: Alpha—α (red), Beta/Mu—β/μ (blue), Gamma/Lambda—γ/λ (black), Delta—δ (orange), and Omicron—ο (green). Within each box plot, orange bars represent the median codon frequency of each clade distribution, red dots represent outliers, and black boxes represent the first and third quartiles (often hidden behind the orange median bar). **A** Spike gene GAT (aspartic acid) frequency distributions (selected months) (box plots) and the average GAT frequency over time (line graph). **B** E gene CTG (leucine) frequency distributions (selected months) and average CTG over time. **C** M gene TAC (tyrosine) frequency distributions (selected months) and average TAC over time. **D** N gene CGA (arginine) frequency distributions (selected months) and average CGA over time. All line graphs utilized the bold regions of the Table 1 clade distributions

Codon distribution plots for each gene highlighted in Fig. 3 and in the supplemental information were chosen based on the greatest number of significant differences between clades summed over 32 months (Additional file 7: Table S7, Additional file 22: Figure S10, Additional file 23: Figure S11, and Additional file 24: Figure S12). Individual months depicted as box plots in Fig. 3 for each clade are examples spanning the most heavily sampled time periods with all clades present (Table 1). The line plots in Fig. 3 show the average codon frequency for each clade over the most heavily sampled months specific to each clade (bolded values in Table 1).

A codon frequency data analysis of the codon GAT (aspartic acid) in the Spike gene, CTG (leucine) in the E gene, TAC (tyrosine) in the M gene, and CGA (arginine) in the N gene showed very low variation (Fig. 3). However, similar to nucleotide frequency distributions, statistical analyses on these codon frequency data showed that many of the clade comparisons are significantly different. Statistical results for these specific codons are summarized in Table 4 and all the data can be found in Additional file 7: Table S7 and Additional file 8: Table S8. The greatest number of significant differences between clade nucleotide distributions for Spike, E, M, and N genes resulted in overlapping months in the spring and summer of 2021, though similar in number (Table 4). Codon usage analysis for non-structural genes also resulted in low variation across and within clades over time (Additional file 9: Table S9, Additional file 10: Table S10, Additional file 23: Figure S11, and Additional file 24: Figure S12).

**Table 4:** SARS-CoV-2 Structural Gene Codon Significant Difference Analysis Highlighting Specific Months

Gene: Codon	*Spike: GAT*	*E: CTG*	*M: TAC*	*N: CGA*
Month(s) with greatest number of significant differences (summed across all clades)	April, June, and July 2021: 9 differences each	March 2021: 9 differences	May and June 2021: 8 differences each	April and July 2021: 9 differences each
Range of significant *p*-values (order corresponding to month in cell above)	7.01E^−07^-0 5.17E^−06^-0 1.12E^−06^-0	3.27E^−08^-0	3.11E^−08^-0 2.85E^−06^-0	4.31E^−05^-0 1.65E^−09^-0
Adjusted alpha (order corresponding to month in first cell)	8.35E^−05^ 8.33E^−05^ 8.36E^−05^	1.47E^−04^	9.14E^−05^ 9.01E^−05^	8.43E^−05^ 8.43E^−05^

Data from which this table was created can be found in Additional file 7: Table S7 and Additional file 8: Table S8

**Fig. 4:**
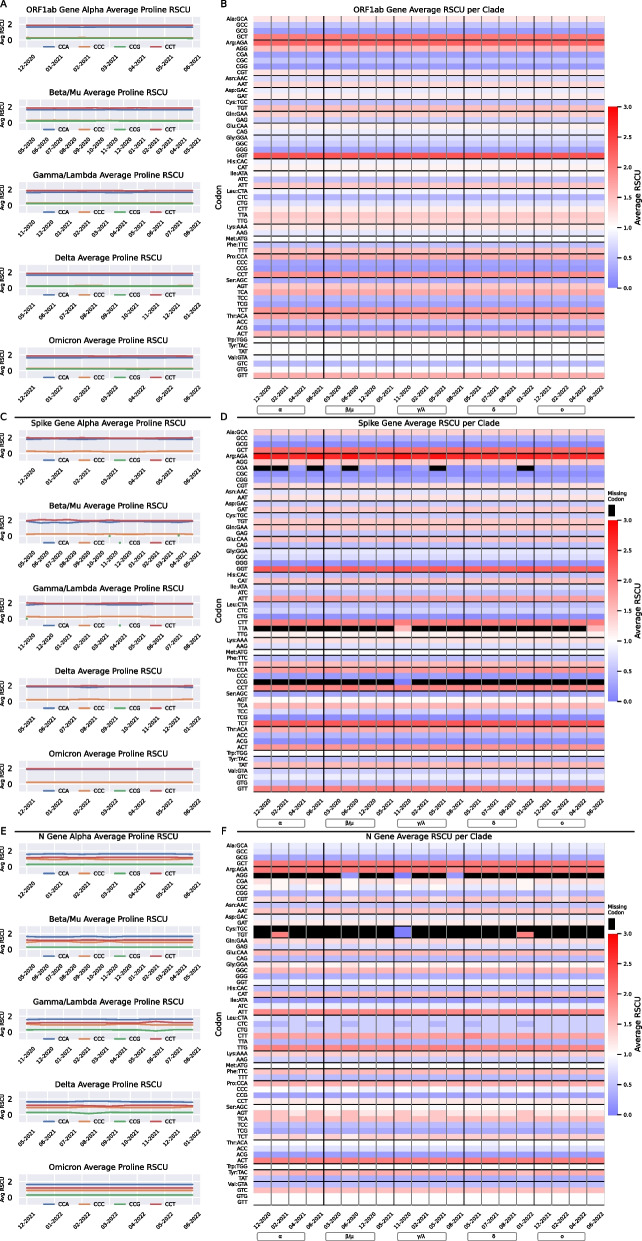
Proline Usage Remains Stable Over Time Across Clades—AGA (Arg) Consistently Overrepresented in ORF1ab, Spike and N Genes. Line graphs highlight the average relative synonymous codon usage (RSCU) of the four proline codons (CCA (blue), CCC (orange), CCG (green), and CCT (red)). Heatmaps show the average RSCU per codon (y-axis) for four months spanning each clades largest sampling months (bolded regions of Table 1). A codon with a RSCU value of > 1.5 is considered highly overrepresented and < 0.5 is considered highly underrepresented. Black boxes represent a missing codon. **A** ORF1ab gene proline average RSCU over time. **B** ORF1ab gene average RSCU for all codons. **C** Spike gene proline average RSCU over time. **D** Spike gene average RSCU for all codons. **E** N gene proline average RSCU over time. **F** N gene average RSCU for all codons. All line graphs utilized the bold regions of the Table 1 clade distributions

After observing very low codon usage distribution changes over time for each of the five clades, we sought to further examine codon bias by calculating the relative synonymous codon usage (RSCU) across the 3,573,491 SARS-CoV-2 sequences. We focused our analysis on the three genes consisting of at least 1000 nucleotides (ORF1ab, Spike, and N genes shown in Fig. 4) and ignored shorter genes for which it is more likely that a codon is represented just by chance. Also highlighted in Fig. 4 is proline average RSCU over the largest sampling months for each clade (bold distributions in Table 1). We found a consistent bias of proline codons towards CCT and CCA, followed by CCC and CCG for ORF1ab sequences of all clades (Fig. 4A). When we evaluate all codons across four months spanning each clade’s greatest sampling months, very minor difference in proline average RSCU between clades was observed. Specifically, Fig. 4B shows that GCT (alanine), AGA (arginine), and GGT (glycine) are consistently the top three overrepresented (> > 1.5) and GCG (alanine), CGG (arginine), and CCG (proline) are consistently the top three underrepresented codons (< < 0.5) for ORF1ab gene. The Spike gene shows a similar bias among all clades towards CCT and CCA proline codons, while CCG is used minimally (Fig. 4C). Calculation of the average RSCU for each clade spanning their largest sampling months (bolded months in Table 1) revealed that Spike gene sequences are highly biased towards AGA (arginine), GGT (glycine), and TCT (serine) codons (top three >  > 1.5), and nominally use GCG (alanine), CCG (proline), and TCG (serine) codons (bottom three <  < 0.5) (Fig. 4D).

We observed differences in comparison to ORF1ab and Spike genes in the average RSCU trends among the N gene sequences. Though, the average RSCU for proline codons are similar across all clades; CCA is used more often than CCT, and there is no substantial separation in usage between CCA, CCT and CCC, CCG (Fig. 4E). The average RSCU fluctuated over time for several codons within each clade (Fig. 4F). The top three highly overrepresented codons (> > 1.5) in N gene sequences are GCT (alanine), AGA (arginine), and ACT (threonine), and the bottom three underrepresented codons (< < 0.5) are AGG (arginine), TGC (cysteine), and ATA (isoleucine) (Fig. 4F).

### CAI and dN/dS over time

**Fig. 5:**
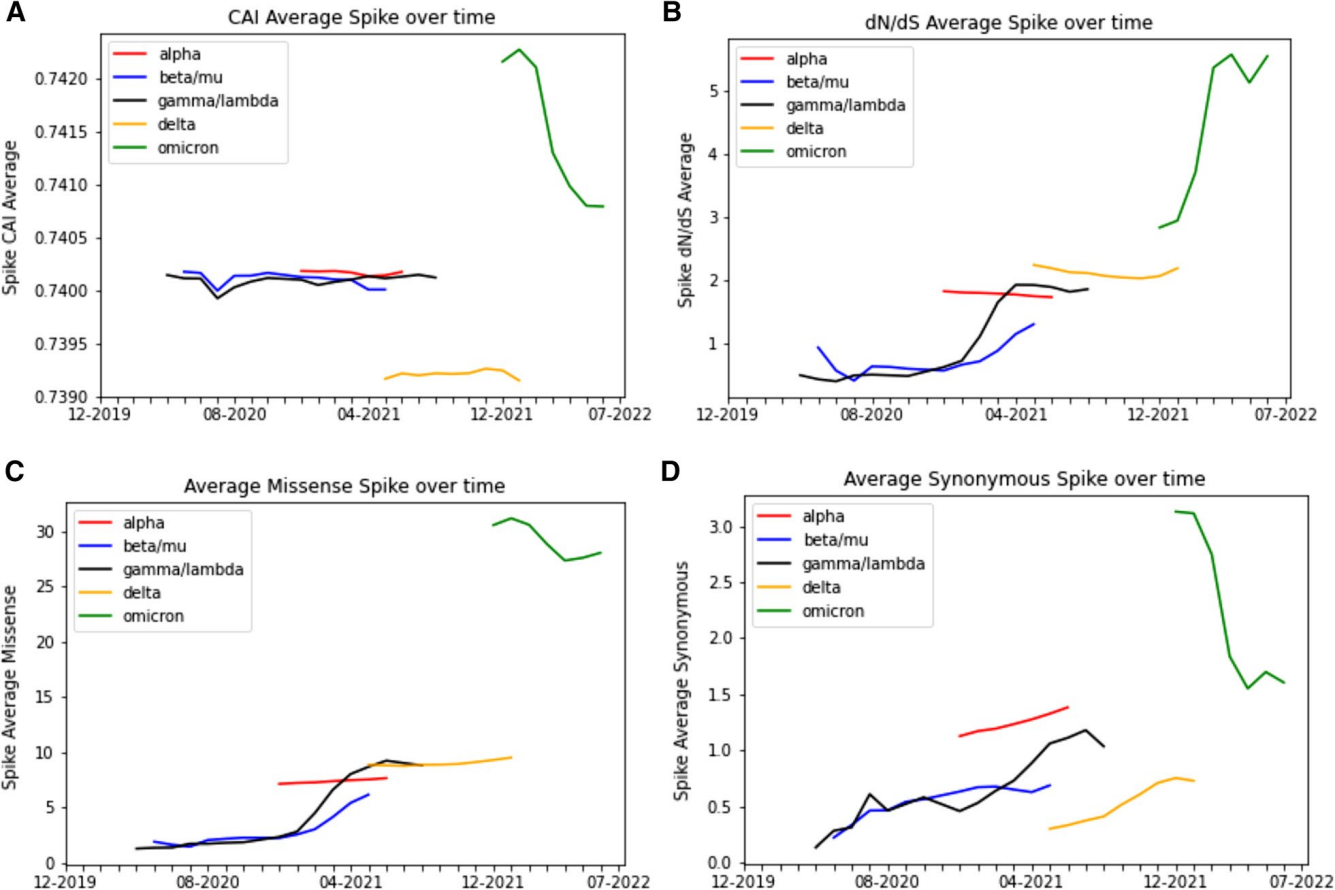
CAI Decreases While dN/dS Increases at Later Timepoints. For all panels, only the months in the bold italic highlighted portion of Table 1 are displayed. Clades are represented by their Greek letter and corresponding color for line graphs: Alpha—α (red), Beta/Mu—β/μ (blue), Gamma/Lambda—γ/λ (black), Delta—δ (orange), and Omicron— (green). **A** Average CAI over time. **B** Average dN/dS over time. **C** Average synonymous mutations per sequence over time. **D** Average missense mutations per sequence over time

In order to measure how similar SARS-CoV-2 Spike gene codon usage is to human codon usage, we calculated CAI as it changed over time. Separately, we also calculated dN/dS over time in order to measure whether or not nonsynonymous changes were favored at specific months. We plotted both these values in Fig. 5 for each clade for the months highlighted in bold italic in Table 1, along with average synonymous mutations per sequence and average missense mutations per sequence (Table 5). For Alpha, Beta/Mu, Gamma/Lambda, and Delta, the CAI remains largely constant over their respective timespans, with peak CAI values occurring towards the latter end of the various timepoints (Alpha and Omicron are an exception) (Fig. 5A). For Omicron, a dramatic decrease in CAI is observed from its onset compared to its final timepoint, though its CAI values are higher in comparison to other clades. The peak CAI values for Alpha, Beta/Mu, Gamma/Lambda, Delta, and Omicron were 0.7402 (December 2021), 0.7402 (November 2020), 0.7402 (July 2021), 0.7393 (November 2021), and 0.7423 (January 2022), respectively, compared to the reference CAI of 0.7405 (Fig. 5A).

**Table 5:** Total Number of Mutations for each Virus, Divided by Synonymous and Nonsynonymous

Virus—strain/clade	Total missense mutations	Total synonymous mutations	Date range	Number of sequences
SARS-CoV-2, Spike- Alpha	8,226,073	1,358,712	12/2019–02/2022	496,938
SARS-CoV-2, Spike—Beta/mu	1,721,205	382,138	12/2019–04/2022	207,758
SARS-CoV-2, Spike—Delta	27,031,401	1,983,386	12/2019–06/2022	1,408,083
SARS-CoV-2, Spike—Gamma/Lambda	3,138,059	662,222	12/2019–05/2022	264,741
SARS-CoV-2, Spike—Omicron	46,728,781	9,935,369	12/2019–07/2022	1,195,971
Influenza, HA- H1N1	427,034	664,436	01/1989–02/2019	15,080
Influenza, HA—H3N2	841,792	1,375,395	04/1982–06/2021	29,045
Dengue virus, E—Dengue 1	24,369	163,741	10/2019–01/2005	1676
MERS-CoV, Spike	1191	6660	12/2008–04/2019	531

The dN/dS rises over time for four out of five clades, most notably for Gamma/Lambda, Beta/Mu and Omicron and less so for Delta (Fig. 5B). Alpha exhibited a decrease in dN/dS over time. As with CAI, the dN/dS were much higher for Omicron at any timepoint when compared to the other clades. The max dN/dS for Alpha, Beta/Mu, Delta, Gamma/Lambda, and Omicron were 1.815 (December 2020), 1.295 (May 2021), 1.917 (April 2021), 2.234 (May 2021), and 5.79 (April 2022) (Fig. 5B).

We also evaluated trends of CAI and dN/dS for genes other than Spike (Additional file 25: Figure S13 and Additional file 26: Figure S14, respectively), and these trends seemed to vary considerably depending on the gene and clade. For the structural proteins, peaks and valleys were observed for the different months, though all CAI values tend to remain within 0.01 of each other across time (Additional file 25: Figure S13). Similar to CAI, the dN/dS trends are variable depending on the gene. Among the four structural protein coding genes, Spike and N have the most dramatic changes over time, with M and E demonstrating less dramatic changes (Additional file 25: Figure S13A, C, B and D, respectively). Most of the nonstructural proteins (apart from ORF3a, ORF7a and ORF8 genes) demonstrate relatively small changes over time, and all of the nonstructural proteins have far less dramatic changes than Spike gene (Additional file 26: Figure S14).

### Mutation heatmaps

**Fig. 6:**
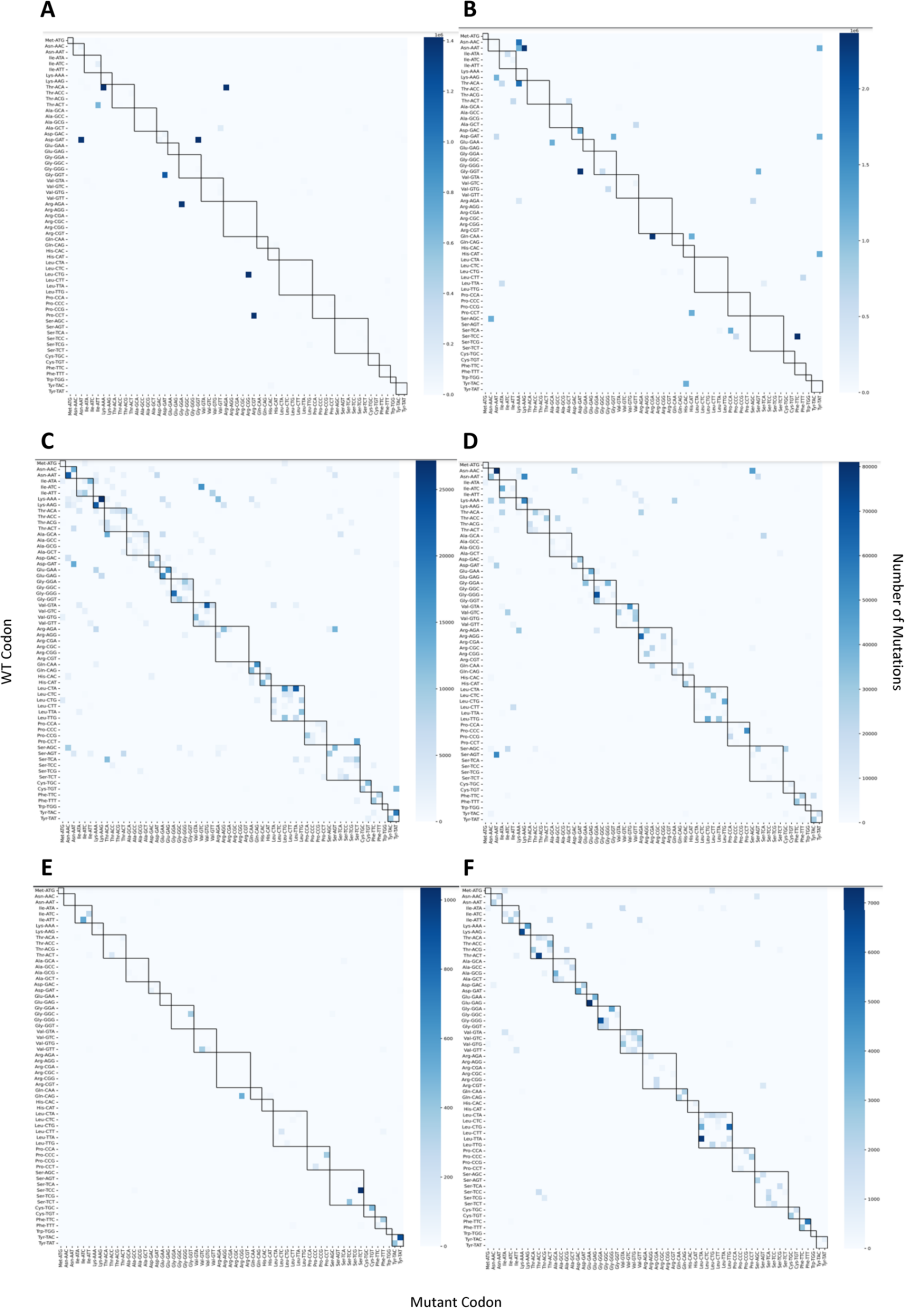
Nonsynonymous Mutations are Greatly Preferred in SARS-CoV-2 Omicron and Delta when Compared to Other Viruses. Both axes represent individual codons, with each point on the heatmap representing a mutation from one codon to the other. Darker points on the heatmap represent more frequent mutations. Mutations within the black boxes are synonymous, and mutations outside these boxes are nonsynonymous. Individual panels show heatmaps for **A** 1,407,663 SARS-CoV-2 Delta Spike Protein sequences ranging from 10/2020 to 06/2022, **B** 1,195,200 SARS-CoV-2 Omicron Spike protein sequences ranging from 11/2021 to 07/2022, **C** 15,080 H1N1 HA protein sequences ranging from 01/1989 to 02/2019, **D** 29,045 H3N2 HA sequences ranging from 12/1980 to 06/2022, **E** 531 MERS-CoV Spike Protein Sequences ranging from 12/2008 to 04/2019, and **F** 1676 Dengue 1 E Protein Sequences ranging from 01/2005 to 10/2019

To visualize the number of each type of mutation (grouped broadly into synonymous and nonsynonymous), we plotted each type of codon substitution (e.g., AAT to AAC, TAC to TAT, etc.) on heatmaps, with darker colors indicating a higher proportion of a given mutation (Fig. 6). We specifically selected viruses that share a similar nucleic acid profile to SARS-CoV-2 (RNA viruses) and selected protein coding regions for genes similar to Spike (related to cell entry or fusion). The relative proportion of nonsynonymous mutations is highest in both Delta and Omicron SARS-CoV-2 strains, followed by H1N1, H3N2, Dengue 1, and MERS-CoV. Each plot in Fig. 6 represents the totality of the sequences we collected for each virus. As these viruses each had different numbers of available sequences, we decided to see whether the number of sequences sampled would alter our conclusions. In Additional file 27: Figure S15, we control for the number of sequences in our SARS and Influenza samples and show that it is minimally altered from the original visualization in Fig. 6 with the total cohort of sequences. In order to confirm that these mutation visualizations were not being significantly biased by mutations that were found in less than 2 sequences, we also generated heatmaps that excluded these rare mutations, to be found in Additional file 28: Figure S16, which resulted in findings similar to heatmaps that had no mutations excluded. Based on this, our conclusions remain the same.

Next, we incorporated information about the position of the codon changes along the Spike sequence into the presentation of our data. Using the same dataset and considering where in the sequence mutations were occurring, we plotted codon and nucleotide composition as heatmaps in Additional file 29: Figure S17. Here, we calculated entropy [42] for every position along the Spike sequence and excluded any sites for which entropy was lower than 0.1. This enabled us to focus on areas along the sequence that had more variable nucleotide or codon composition. Based on the Uniprot annotation for the Spike sequence [43], the receptor binding domain (RBD) is between amino acids 319–541. For SARS-CoV-2 Delta, 5 of the high entropy positions are within the RBD, with the remainder 17 being outside of this region. For many of these positions, the dominant codon is still the WT codon. For SARS-CoV-2 Omicron, there are a higher number of entropy positions [32], with 16 of them within the RBD, indicating that there is a greater proportion of high entropy positions for which the WT codon is not dominant when compared to Delta. For most of the positions along Spike gene for both Delta and Omicron, nonsynonymous mutations outnumber synonymous mutations—however, there are exceptions, such as positions 155, 409, 412, 476, 573, 855, 881, 1121, and 1258 for Delta, and positions 10, 52, 67, 409, and 451 for Omicron (Table 6). A full accounting of all the mutations within our dataset can be found in Additional file 11: Tables S11-70.zip. Further, we have added Uniprot annotations to all of the mutations for structural proteins in Additional file 12: Tables S71-90.zip, to give insight onto regions and functional domains associated with particular mutations.

**Table 6:** Wild Type, Synonymous, and Nonsynonymous Codons per High Entropy Position (exceeding 0.1) on SARS-CoV-2 Spike

SARS-CoV-2 delta				SARS-CoV-2 omicron			
Position	WT codons	Synonymous codons	Nonsynonymous codons	Position	WT codons	Synonymous codons	Nonsynonymous codons
4	1,387,485	51	20,255	10	1,071,175	124,747	40
94	792,111	88	615,597	18	593,973	16	601,961
111	1,374,841	642	32,333	23	598,455	29	246,641
141	220,153	4	1,186,783	52	1,111,530	84,384	36
144	1,368,800	1854	36,124	66	606,936	39	588,890
155	21,903	191	150	67	553,440	542,664	3785
156	21,572	94	123	94	605,813	18	589,846
157	21,145	6	1,378,971	141	8899	194	1,140,676
221	1,253,151	46	154,877	211	609,770	234	540,403
288	1,389,455	57	18,564	212	597,049	41	598,862
409	1,383,411	24,569	101	338	11,212	0	1,184,745
412	1,386,531	21,428	122	345	823,247	27	372,129
476	1,393,255	8165	6652	370	13,942	5	1,182,006
573	1,367,400	38,500	2180	375	598,382	26	597,529
612	1,388,221	56	19,803	404	600,131	15	595,804
855	1,371,204	35,927	944	407	603,498	26	592,427
881	1,300,777	107,237	61	409	1,065,180	130,764	10
949	30,891	1	1,377,121	416	50,767	0	1,145,194
1103	1,389,498	118	18,462	439	60,885	1	1,135,053
1121	1,383,517	23,035	1529	445	636,351	1	559,592
1258	1,362,896	39,980	5204	451	1,048,422	274	147,250
1263	1,371,670	2790	33,609	483	14,073	0	1,181,087
				485	1,146,988	27	48,153
				492	61,473	3	1,133,675
				495	610,353	29	584,748
				504	15,157	3	1,179,979
				546	607,050	37	588,874
				700	1,157,571	58	38,333
				703	1,100,568	34	95,357
				763	21,025	40	1,174,898
				855	605,800	1207	588,952
				980	607,167	10	588,784

## Discussion

In this study, we analyzed the nucleotide and codon frequency data for 3,573,491 SARS-CoV-2 sequences, covering 12 genes (ORF1ab, Spike, ORF3a, ORF3b, E, M, ORF6, ORF7a, ORF7b, ORF8, N, and ORF10) and five GISAID specified clades (Alpha, Beta/Mu, Gamma/Lambda, Delta, and Omicron) over 32 months (December 2019–July 2022) [28]. We discovered when analyzing each gene individually there is very minimal overall nucleotide and codon variation between and within clades over time regardless of the length of the gene. We subsequently showed that within this pool of few mutations, nonsynonymous Spike gene mutations overwhelmingly dominate the phenotypic landscape with relatively strong selection pushing for changes away from human codon usage trends/bias in most cases. Overall, our findings showed that as SARS-CoV-2 spreads around the world, small shifts in nucleotide and codon frequency are enough to produce a variety of clades expressing varying degrees of infectivity and virulence [44].

Several previous studies also looked at nucleotide and codon usage of SARS-CoV-2, but used relatively small sample sizes, covered shorter date ranges, excluded clades, and/or analyzed concatenated genes or full sequences [24–27, 45–49]. By analyzing the genes individually and expanding the number of sequences and dates, we found that most SARS-CoV-2 genes over time favored the T nucleotide followed by A, C and G. Roy et al. [22] found similar results with 99 GISAID concatenated sequences downloaded in February 2020; though, slight variations in nucleotide frequency have been found [23, 45, 47, 50], A/T bias remains the same, except within the N gene (A > C > G > T) (Fig. 2). Low GC content is found in all coronaviruses and has been shown to strengthen translation initiation through biochemical rigidity [50, 51]. The G and/or C nucleotide content is on average suppressed in most clades for the genes analyzed in this study. At the same time, the T nucleotide content on average increased in ORF1ab, ORF3a, ORF3b, E, ORF7b, ORF8, and N genes (Additional file 5: Table S5). ORF6 has the biggest disparity between A/T and G/C frequencies, though this difference is decreasing over time for most clades. The N gene is unique in its nucleotide content; however, there is a gradual increase in the T nucleotide and decrease in the C nucleotide for all clades but Alpha. Several publications have shown evidence for hypermutation C—> T transitions and its possible relation to RNA editing processes via APOBEC proteins [25, 48, 49]. This may be one explanation for the overall maintenance of low GC content of SARS-CoV-2 sequences, despite the large difference from human usage [48].

As Fig. 2 illustrates, we saw very little nucleotide variation within and between clades for each gene. Nonetheless, many of these differences between clades were significant and aided in their divergence (see Methods; Additional file 2: Table S2 and Additional file 3: Table S3). We can zoom in on these distributions and note some interesting observations: (1) average nucleotide frequency fluctuations over time are much smaller than changes in variance (for most genes, variance fluctuates similarly for all clades but Omicron), and (2) the average and variance of these distributions over time are shown to positively covary for Spike, ORF3a, ORF3b, E, ORF6, ORF7a, N, and ORF10 genes, and negatively covary for ORF1ab, M, ORF7b, and ORF8 genes. Many biological analyses focus on following the average over time (even when full distribution data is displayed) [24, 47], but other moments (i.e., properties or characteristics) of the distribution can influence the evolutionary trajectory of a given clade. Following the variance in nucleotide frequency and not just the average is important since large distribution variances tend to reduce the strength of selection; and therefore, reduce the likelihood of a particular nucleotide increasing in frequency over time (everything else held equal) [52]. For most SARS-CoV-2 genes, changes in variance over time for all nucleotides are quite small, though increasing in most cases and peaking in different months for different genes. Selection’s increasingly weak magnitude, independent of direction, has been relatively maintained across these 32 months for SARS-CoV-2. Nonetheless, we documented many significant differences between the clades and their nucleotide distributions over time. We noted in the results that there were more differences in some months over others but did not find any causal reasoning behind it.

Our codon analysis was performed to assess the codon bias within each of the five clades, across 12 genes, and over 32 months. We found that the nucleotide preference within codons does not strictly adhere to the nucleotide bias discussed above for whole genes. For example, the top three overrepresented codons for ORF1ab (GCT, AGA, and GGT) and Spike (AGA, GGT, and TCT) genes use the G nucleotide most often, though the T nucleotide is preferred in the third position of the codon (Fig. 4B, D). The unique nucleotide bias of N gene (A > C > G > T) (Fig. 2G) resulted in a bias towards the A nucleotide within its codons. The T nucleotide is the least used nucleotide across the N gene; nonetheless, it is more likely to reside in the third position among the top overrepresented codons (GCT, AGA, and ACT) (Fig. 4F). Similar RSCU results were found when concatenating genes—codon AGA is found to possess an RSCU value > 2, regardless of date or location of samples [34, 47, 50].

When we look closely at the codon with the highest RSCU value for ORF1ab, Spike and N genes (GGT, AGA, and AGA respectively), there is an overall decrease in the average frequency across most clades over time (Beta/Mu increases for ORF1ab and Spike genes, Alpha increases for Spike gene) (Additional file 9: Table S9). We also found an overall decrease in the frequency variance for ORF1ab (GGT) and N (AGA) genes, and an increase for the Spike (AGA) gene over time (Beta/Mu increases for ORF1ab gene) (Additional file 10: Table S10). This means the SARS-CoV-2 individual codon usage over time looks more alike with fewer GGT codons in their ORF1ab gene and AGA codons in their N gene. Meanwhile, diversity in the use of the Spike gene AGA codon is increasing. This is interesting because the three codons with the highest RSCU values in humans (CTG, GCC, and CAG) are GC heavy, especially in the first and third codon positions. Overall, the SARS-CoV-2 codon usage remains quite different from human usage [53]. This antagonism may aid the SARS-CoV-2 population by promoting better folded proteins [34] and avoiding competition [25].

We narrowed our focus to a specific amino acid group to identify explicit differences between synonymous codons within each clade and across genes ORF1ab, Spike, and N. Proline codon (CCA, CCC, CCG, and CCT) usage was of particular interest to follow because of its slow rate of translation and requirement of a specific elongation factor (eIF5A) for translation of polyproline sequences [54]. ORF1ab, Spike, and N genes showed slightly different proline codon preferences that were maintained over the 32 months for each clade (Fig. 4A, C, E). All three genes for all clades preferred codons CCA and CCT, but the rarely used proline codons are what set these three genes and clades apart. CCG and CCC are regularly used in the ORF1ab gene at similar frequencies, CCC in the Spike gene, and both CCG and CCC in the N gene but at differing frequencies. This codon usage is consistent with the results of the top three RSCU valued codons in ORF1ab, Spike and N genes, where A and T nucleotides are more likely to be present in the third position. It has been shown in bacteria that the synonymous proline codon used matters in the context of translation efficiency [55]. Human proline RSCU (CCC > CCT > CCA > CCG) is quite a bit different than SARS-CoV-2 usage and may further promote the slowing of translation [53].

We also looked more closely at codons for each gene that resulted in the largest total number of significant differences between clades summed over all 32 months (Additional file 7: Table S7 and Additional file 22: Figure S10). Within each month, codon distributions for each clade are compared pairwise, unless sample size is insufficient for the calculation. With very little variation within each clade’s codon distributions over time (as seen in Fig. 3 and Additional file 23: Figure S11 and Additional file 24: Figure S12), we found that this is enough variation to drive significant differences between clades. There were a few interesting observations of these codons (ORF1ab—CTT, Spike—GAT, ORF3a—CAG, ORF3b—TTA, E—CTG, M—TAC, ORF6—GAC, ORF7a—GTT, ORF7b—TTG, ORF8—CAA, N—CAG, and ORF10—CTC): (1) they consist of mostly T nucleotides and few G nucleotides, (2) they are more likely to possess a C nucleotide in the first position, equally an A or T nucleotide in the second position, and a G nucleotide in the third position, and (3) CAG (glutamine) is the only codon with the most significant differences found in more than one gene (ORF3a and N). We also analyzed the average and variance over time of these codon frequency distributions and found that most clades of a given gene fluctuate together. We show that within a gene, these codons are more likely to decrease in average and variance over time (Additional file 9: Table S9 and Additional file 10: Table S10). As we mentioned when discussing nucleotide frequency, following the variance in these codon frequency distributions matters. Reducing the variance in codon frequency increases the magnitude of selection [52]; and therefore, the direction selection pushes the SARS-CoV-2 population may not change much, but the step size at which it does so can potentially aid the divergence of the SARS-CoV-2 clades.

Our CAI analysis shows that SARS-CoV-2 codon usage similarity to the host does change over time, though only slightly in most cases, and demonstrates that trends are not identical for different genes (Fig. 5 and Additional file 25: Figure S13). Spike gene, which is considered an important and consequential antigenic site for the virus [56], had fairly consistent CAI values for most clades, but a dramatic drop in CAI for Omicron, indicating that more recent (and contagious) strains of the virus are moving away from human codon usage. No other gene mimics this trend for Omicron (the closest being ORF6 gene, though this still shows an increase in CAI in later months), which could point towards a unique codon usage characteristic of the Spike gene. Posani et al*.* calculated and compared CAI for several genes, including Spike gene, from December 2019 to February 2021, and their primary conclusion was that CAI tends to decrease over time for these genes [8]. Mogro et al*.* calculated changes in CAI from December 2019 to September 2021, finding a negative trend in CAI when all genes are concatenated. However, this trend becomes less apparent when sequences were separated by gene or clade [24]. Their dataset for the dN/dS analysis is considerably more constrained (a total of about 200,000 sequences). Our analysis still concurs with their observation that CAI does not have a consistent downwards trend over time when sequences are separated by gene, and there are notable differences between different clades, genes, and timepoints in CAI [24].

Our dN/dS analysis also finds the Spike gene to be different from the other genes—for Alpha, Delta, and Omicron, the ratio is above one for all time points and for Gamma/Lambda, it is above one for most timepoints after 2021. This shows that nonsynonymous changes are favored within the Spike gene for these clades and timepoints. There are no other genes which have any clades with dN/dS consistently above one other than ORF8 gene Alpha, which is above 1 for a few timepoints in early 2021. This is yet another way in which Spike gene’s mutation profile differs from other SARS-CoV-2 genes, which could be connected to its role as the primary cell entry mediator for the virus. Nikolaidis et al*.* performed an analysis calculating dN/dS over time for about 850,000 sequences (mixed NCBI and GISAID data) up till December 2021 for Spike, ORf1a, and ORF1b genes; they note that dN/dS had started to increase notably for Spike gene in the second year of the pandemic, though still remained relatively close to one. The average dN/dS ratio for Spike gene was noticed to be above one for all clades [57]. This is fairly consistent with our results, though their dN/dS numbers are lower than ours for Spike gene at similar time points. Of note, our data includes Omicron within the first half of 2022, during which dN/dS increased far more than any previous time point. We also show data for genes not featured in the aforementioned study—all of the dN/dS values for these genes are far lower than that of Spike gene.

Visualization of the synonymous and nonsynonymous mutations of SARS-CoV-2 using heatmaps supported our dN/dS calculations for Spike gene, showing that nonsynonymous mutations far outnumber synonymous ones for both Omicron and Delta. This contrasts with other RNA viruses, which show comparatively fewer nonsynonymous mutations. In a clinical study investigating intra-host single-nucleotide variants (iSNVs), nonsynonymous substitutions were noted to be overrepresented, indicating that there is a biological phenomenon at play in determining the synonymous and nonsynonymous mutations observed for this virus [58–60]. One factor that could be relevant to this observed difference between SARS-CoV-2 and other RNA viruses is the mechanisms underlying the fidelity of viral polymerases and the absence/presence of the associated proofreading activities. Literature sources indicate that the Dengue virus RNA-Dependent RNA polymerase (RdRp) has an error rate of approximately 1e^−4^ mutations per base pair [58–60], Influenza A RNA polymerase has an error rate of approximately 1.5e^−5^ mutations per base pair [61], and SARS-CoV-2 RdRp has an error rate between 1e^−6^ and 5e^−6^ mutations per base pair [62] (estimations for the MERS-CoV mutation rate exist, but they do not use comparable methodologies to the ones cited here for the other viruses). SARS-CoV-2’s substantially lower error rate than the other RNA viruses points to a different proofreading mechanism, which could be linked to the preponderance of nonsynonymous mutations—it is possible that this mechanism disfavors synonymous changes. Specifically, SARS-CoV-2 (along with other coronaviruses) encodes nsp14, a 3′–5′ exoribonuclease that ameliorates the poor fidelity of the RdRp [63]—this is encoded within the ORF1ab region of the genome [64]. Influenza A RNA polymerase and Dengue virus RdRp are both considered low fidelity replicases [60, 65]. The additional proofreading mechanism in SARS-CoV-2 could be tied to the difference observed in synonymous and nonsynonymous mutations when compared to other RNA viruses—though this would require further investigation to confirm.

We further show the codon and nucleotide composition of SARS-CoV-2 Spike Omicron and Delta in Additional file 29: Figure S17, and tabulate the portion of synonymous, nonsynonymous, and WT codons in Table 6. Together, these data support the notion that nonsynonymous changes far outnumber synonymous ones for SARS-CoV-2, but this is highly position-dependent, and at certain locations along the Spike sequence, synonymous changes are overwhelmingly dominant. Altogether, this could point towards an underlying biological mechanism in SARS-CoV-2 that favors nonsynonymous changes in most locations, or it could be that SARS-CoV-2 is a relatively “recent” virus (at least, compared to the other viruses featured in our study), and might show a different synonymous/nonsynonymous mutation profile later in its evolution.

## Conclusion

This study presents a multifaceted analysis of more than 3.5 million GISAID SARS-CoV-2 sequences since the beginning of the 2019 pandemic. We have demonstrated differences in the contribution of each of the SARS-CoV-2 genes and their clades to the overall population, and some unique qualities not shared with other RNA viruses. Such retrospective analysis of SARS-CoV-2 viral genomes can yield valuable data that could inform future vaccine development through strategies like viral deoptimization.

This research has highlighted the minor, but in many cases statistically significant, differences in SARS-CoV-2 clade nucleotide and codon usage over time. Nonetheless, our results show a notable difference in the variance of those distributions, which in turn may heavily influence the direction of evolution. Currently, we are looking into how location specific traits along the Omicron Spike gene contribute to the overall fitness of the virus over time. This bottom-up approach may lead to a specific set of traits associated with Omicron sub-lineages.

## Supplementary Information

**Additional file 1**. **Table S1**: Nucleotide Frequency Variance.

**Additional file 2**. **Table S2**: Nucleotide Significance Data.

**Additional file 3**.** Table S3**: Nucleotide Significant Differences Summary.

**Additional file 4**.** Table S4**: Average AG/TC Frequency.

**Additional file 5**.** Table S5**: Average AT/GC Frequency.

**Additional file 6**.** Table S6**: Average Nucleotide Frequency.

**Additional file 7**.** Table S7**: Codon Significant Differences Summary.

**Additional file 8**.** Table S8**: Codon Significance Data.

**Additional file 9**.** Table S9**: Average Codon Frequency.

**Additional file 10**. **Table S10**: Codon Frequency Variance.

**Additional file 11**.** Tables S11-70**: Counts of All Mutations for each Gene/Clade Combination.

**Additional file 12**. **Tables S71-90**: Uniprot Annotations for All Mutations in Structural Proteins for all Clades.

**Additional file 13**.** Figure S1**: Greatest Number of Significant Differences Found Between N Gene Nucleotide Frequency Distributions. Each column represents the sum of the number of significant differences resulting from comparisons between clades for each nucleotide frequency distribution per month (December 2019–July 2022). Significance was determined based on a Bonferroni corrected alpha threshold, scaled by the number of tests ran per month (Additional file 7: Table S7).

**Additional file 14**.** Figure S2**: Monthly Snapshots Reveal Minimal Variation in the AT/GC Frequency Distributions. Clades are represented by their Greek letter and corresponding color for line graphs: Alpha—α (red), Beta/Mu – β/μ (blue), Gamma/Lambda—γ/λ (black), Delta—δ (orange), and Omicron—ο (green). Within each box plot, orange bars represent the median AT/GC frequency of each clade distribution, red dots represent outliers, and black boxes represent the first and third quartiles (often hidden behind the orange median bar). A) Spike gene AT/GC frequency distributions over selected months. B) Spike gene average AT/GC frequencies plotted over significantly large sampling months. C) E gene AT/GC frequency distributions over selected months. D) E gene average AT/GC frequencies plotted over significantly large sampling months. E) M gene AT/GC frequency distributions over selected months. F) M gene average AT/GC frequencies plotted over significantly large sampling months. G) N gene AT/GC frequency distributions over selected months. H) N average AT/GC frequencies plotted over significantly large sampling months. All line graphs utilized the bold regions of the Table 1 clade distributions. Data found in Additional file 5: Table S5.

**Additional file 15**.** Figure S3**: Purine/Pyrimidine (AG/TC) Frequency Distributions are Consistent Across Clades and Time. Clades are represented by their Greek letter and corresponding color for line graphs: Alpha—α (red), Beta/Mu—β/μ (blue), Gamma/Lambda—γ/λ (black), Delta—δ (orange), and Omicron—ο (green). Within each box plot, orange bars represent the median AG/TC frequency of each clade distribution, red dots represent outliers, and black boxes represent the first and third quartiles (often hidden behind the orange median bar). A) Spike gene AG/TC frequency distributions over selected months. B) Spike gene average AG/TC frequencies plotted over significantly large sampling months. C) E gene AG/TC frequency distributions over selected months. D) E gene average AG/TC frequencies plotted over significantly large sampling months. E) M gene AG/TC frequency distributions over selected months. F) M gene average AG/TC frequencies plotted over significantly large sampling months. G) N gene AG/TC frequency distributions over selected months. H) N gene average AG/TC frequencies plotted over significantly large sampling months. All line graphs utilized the bold regions of the Table 1 clade distributions. Data found in Additional file 4: Table S4.

**Additional file 16**.** Figure S4**: Nucleotide Frequency Distributions Over Time per Clade in ORF1ab, ORF3a, ORF3b and ORF6 Genes Show Little Change Over Time. Clades are represented by their Greek letter and corresponding color for line graphs: Alpha—α (red), Beta/Mu—β/μ (blue), Gamma/Lambda—γ/λ (black), Delta—δ (orange), and Omicron—ο (green). Within each box plot, orange bars represent the median nucleotide frequency of each clade distribution, red dots represent outliers, and black boxes represent the first and third quartiles (often hidden behind the orange median bar). A) ORF1ab gene nucleotide frequency distributions over selected months. B) ORF1ab gene average nucleotide frequencies plotted over significantly large sampling months. C) ORF3a gene nucleotide frequency distributions over selected months. D) ORF3a gene average nucleotide frequencies plotted over significantly large sampling months. E) ORF3b gene nucleotide frequency distributions over selected months. F) ORF3b gene average nucleotide frequencies plotted over significantly large sampling months. G) ORF6 gene nucleotide frequency distributions over selected months. H) ORF6 gene average nucleotide frequencies plotted over significantly large sampling months. All line graphs utilized the bold regions of the Table 1 clade distributions. Data found in Additional file 6: Table S6.

**Additional file 17**. **Figure S5**: Outliers Increase within Nucleotide Frequency Distributions Over Time per Clade in ORF7a, ORF7b, ORF8 and ORF10 Genes. Clades are represented by their Greek letter and corresponding color for line graphs: Alpha—α (red), Beta/Mu—β/μ (blue), Gamma/Lambda—γ/λ (black), Delta—δ (orange), and Omicron—ο (green). Within each box plot, orange bars represent the median nucleotide frequency of each clade distribution, red dots represent outliers, and black boxes represent the first and third quartiles (often hidden behind the orange median bar). A) ORF7a gene nucleotide frequency distributions over selected months. B) ORF7a gene average nucleotide frequencies plotted over significantly large sampling months. C) ORF7b gene nucleotide frequency distributions over selected months. D) ORF7b gene average nucleotide frequencies plotted over significantly large sampling months. E) ORF8 gene nucleotide frequency distributions over selected months. F) ORF8 gene average frequencies plotted over significantly large sampling months. G) ORF10 gene nucleotide frequency distributions over selected months. H) ORF10 gene average nucleotide frequencies plotted over significantly large sampling months. All line graphs utilized the bold regions of the Table 1 clade distributions. Data found in Additional file 6: Table S6.

**Additional file 18**. **Figure S6**: Relatively Large Number of AT/GC Outliers Present Across Time per Clade in ORF1ab Relative to ORF3a, ORF3b and ORF6 Genes. Clades are represented by their Greek letter and corresponding color for line graphs: Alpha—α (red), Beta/Mu—β/μ (blue), Gamma/Lambda—γ/λ (black), Delta—δ (orange), and Omicron—ο (green). Within each box plot, orange bars represent the median AT/GC frequency of each clade distribution, red dots represent outliers, and black boxes represent the first and third quartiles (often hidden behind the orange median bar). A) ORF1ab gene AT/GC frequency distributions over selected months. B) ORF1ab gene average AT/GC frequencies plotted over significantly large sampling months. C) ORF3a gene AT/GC frequency distributions over selected months. D) ORF3a gene average AT/GC frequencies plotted over significantly large sampling months. E) ORF3b gene AT/GC frequency distributions over selected months. F) ORF3b gene average AT/GC frequencies plotted over significantly large sampling months. G) ORF6 gene AT/GC frequency distributions over selected months. H) ORF6 gene average AT/GC frequencies plotted over significantly large sampling months. All line graphs utilized the bold regions of the Table 1 clade distributions. Data found in Additional file 5: Table S5.

**Additional file 19**.** Figure S7**: Relatively Large Number of AT/GC Outliers Present Across Time per Clade in ORF7a, ORF7b, ORF8 and ORF10 Genes. Clades are represented by their Greek letter and corresponding color for line graphs: Alpha—α (red), Beta/Mu—β/μ (blue), Gamma/Lambda—γ/λ (black), Delta—δ (orange), and Omicron—ο (green). Within each box plot, orange bars represent the median AT/GC frequency of each clade distribution, red dots represent outliers, and black boxes represent the first and third quartiles (often hidden behind the orange median bar). A) ORF7a gene AT/GC frequency distributions over selected months. B) ORF7a gene average AT/GC frequencies plotted over significantly large sampling months. C) ORF7b gene AT/GC frequency distributions over selected months. D) ORF7b gene average AT/GC frequencies plotted over significantly large sampling months. E) ORF8 gene AT/GC frequency distributions (selected months) show a similar pattern over time for each clade AT>GC. F) ORF8 gene average AT/GC frequencies plotted over significantly large sampling months. G) ORF10 gene AT/GC frequency distributions over selected months. H) ORF10 gene average AT/GC frequencies plotted over significantly large sampling months. All line graphs utilized the bold regions of the Table 1 clade distributions. Data found in Additional file 5: Table S5.

**Additional file 20**.** Figure S8**: Minimal Change Over Time Within and Between Clades in Purine/Pyrimidine (AG/TC) Frequency Distributions for ORF1ab, ORF3a, ORF3b and ORF6 Genes. Clades are represented by their Greek letter and corresponding color for line graphs: Alpha—α (red), Beta/Mu—β/μ (blue), Gamma/Lambda—γ/λ (black), Delta—δ (orange), and Omicron—ο (green). Within each box plot, orange bars represent the median AG/TC frequency of each clade distribution, red dots represent outliers, and black boxes represent the first and third quartiles (often hidden behind the orange median bar). A) ORF1ab gene AG/TC frequency distributions over selected months. B) ORF1ab gene average AG/TC frequencies plotted over significantly large sampling months. C) ORF3a gene AG/TC frequency distributions over selected months. D) ORF3a gene average AG/TC frequencies plotted over significantly large sampling months. E) ORF3b gene AG/TC frequency distributions over selected months. F) ORF3b gene average AG/TC frequencies plotted over significantly large sampling months. G) ORF6 gene AG/TC frequency distributions over selected months. H) ORF6 gene average AG/TC frequencies plotted over significantly large sampling months. All line graphs utilized the bold regions of the Table 1 clade distributions. Data found in Additional file 4: Table S4. 

**Additional file 21**.** Figure S9**: Purine/Pyrimidine (AG/TC) Frequency Distributions Consistent Across Clades and Time for ORF7a, ORF7b, ORF8 and ORF10 Genes. Clades are represented by their Greek letter and corresponding color for line graphs: Alpha—α (red), Beta/Mu—β/μ (blue), Gamma/Lambda—γ/λ (black), Delta—δ (orange), and Omicron—ο (green). Within each box plot, orange bars represent the median AG/TC frequency of each clade distribution, red dots represent outliers, and black boxes represent the first and third quartiles (often hidden behind the orange median bar). A) ORF7a gene AG/TC frequency distributions over selected months. B) ORF7a gene average AG/TC frequencies plotted over significantly large sampling months. C) ORF7b gene AG/TC frequency distributions over selected months. D) ORF7b gene average AG/TC frequencies plotted over significantly large sampling months. E) ORF8 gene AG/TC frequency distributions over selected months. F) ORF8 gene average AG/TC frequencies plotted over significantly large sampling months. G) ORF10 gene AG/TC frequency distributions over selected months. H) ORF10 gene average AG/TC frequencies plotted over significantly large sampling months. All line graphs utilized the bold regions of the Table 1 clade distributions. Data found in Additional file 4: Table S4.

**Additional file 22**.** Figure S10**: The Majority of SARS-CoV2 Codon Frequency Distributions Share > 40 Significant Differences Between Clades Across 32 Months. Each column represents the sum of the number of significant differences resulting from comparisons between clades for each codon frequency distribution per month (December 2019–July 2022). Significance was determined based on a Bonferroni corrected alpha threshold, scaled by the number of tests ran per month. Data found in Additional file 7: Table S7.

**Additional file 23**.** Figure S11**: Codon Frequency Distributions Show Little Variation Over Time and Across Clades in ORF1ab, ORF3a, ORF3b and ORF6 Genes. Clades are represented by their Greek letter and corresponding color for line graphs: Alpha—α (red), Beta/Mu—β/μ (blue), Gamma/Lambda—γ/λ (black), Delta—δ (orange), and Omicron—ο (green). Within each box plot, orange bars represent the median codon frequency of each clade distribution, red dots represent outliers, and black boxes represent the first and third quartiles (often hidden behind the orange median bar). Codons highlighted here were selected based on greatest number of significant differences between December 2019 and July 2022 (Additional File 7: Table S7). A) ORF1ab gene CCT (proline) frequency distributions over selected months. B) ORF3a gene CAG (glutamine) frequency distributions over selected months. C) ORF3b gene TTA (leucine) frequency distributions over selected months. D) ORF6 gene GAC (aspartic acid) frequency distributions over selected months. All line graphs utilized the bold regions of the Table 1 clade distributions. Data found in Additional file 9: Table S9.

**Additional file 24**.** Figure S12**: Codon Usage of ORF7a, ORF7b, ORF8 and ORF10 Remains Stable Between Clades and Over Time. Clades are represented by their Greek letter and corresponding color for line graphs: Alpha—α (red), Beta/Mu—β/μ (blue), Gamma/Lambda—γ/λ (black), Delta—δ (orange), and Omicron—ο (green). Within each box plot, orange bars represent the median codon frequency of each clade distribution, red dots represent outliers, and black boxes represent the first and third quartiles (often hidden behind the orange median bar). Codons highlighted here were selected based on greatest number of significant differences between December 2019 and July 2022 (Additional file 7: Table S7). A) ORF7a gene GTT (valine) frequency distributions over selected months. B) ORF7b gene TTG (leucine) frequency distributions over selected months. C) ORF8 gene CAA (glutamine) frequency distributions over selected months. D) ORF10 gene CTC (leucine) frequency distributions over selected months. All line graphs utilized the bold regions of the Table 1 clade distributions. Data found in Additional file 9: Table S9.

**Additional file 25**.** Figure S13**: Line Plots Showing Average CAI Over Time for All Genes. Panels A-L refer to Spike, M, N, E, ORF1ab, ORF3a, ORF3b, ORF6, ORF7a, ORF7b, ORF8, and ORF10, respectively. Data is plotted only for the dates that are bolded & italicized in Table 1.

**Additional file 26**.** Figure S14**: Line Plots Showing dN/dS Ratio Over Time for All Genes. Panels A-L refer to Spike, M, N, E, ORF1ab, ORF3a, ORF3b, ORF6, ORF7a, ORF7b, ORF8, and ORF10, respectively. Data is plotted only for the dates that are bolded and italicized in Table 1.

**Additional file 27**.** Figure S15**: Missense and Synonymous Mutations for SARS-CoV-2 Spike and Influenza Virus HA, Normalized for Sequence Count. Both axes represent individual codons, with each point on the heatmap representing a mutation from one codon to the other. Darker points on the heatmap represent more frequent mutations. Mutations within the black boxes are synonymous, and mutations outside these boxes are nonsynonymous. Each panel shows a heatmap generated from 15,000 sequences of A) SARS-CoV-2 Delta Spike ranging from 10/2020-06/2022, B) 15,000 SARS-CoV-2 Omicron Spike ranging from 11/2021-07/2022, C) 15,000 H1N1 HA ranging from 01/1989-02/2019, and D) H3N2 HA ranging from 12/1980-06/2022.

**Additional file 28**.** Figure S16**: Missense and Synonymous Mutations for SARS-CoV-2 Spike, Omicron and Delta, Comparison Between Full Cohort of Mutations and Rare Mutations Excluded. Both axes represent individual codons, with each point on the heatmap representing a mutation from one codon to the other. Darker points on the heatmap represent more frequent mutations. Mutations within the black boxes are synonymous, and mutations outside these boxes are nonsynonymous. Individual panels show heatmaps representing A) 1,407,663 SARS-CoV-2 Delta Spike Protein Sequences ranging from 10/2020-06/2022, B) 1,407,663 SARS-CoV-2 Delta Spike Protein Sequences ranging from 10/2020-06/2022, with mutations that are presenting in 2 or fewer sequences excluded, C) 1,195,200 SARS-CoV-2 Omicron Spike protein sequences ranging from 11/2021-07/2022, and D) 1,195,200 SARS-CoV-2 Omicron Spike protein sequences ranging from 11/2021-07/2022, with mutations that are presenting in 2 or fewer sequences excluded.

**Additional file 29**.** Figure S17**: Missense and Synonymous Mutations at High Entropy Positions Along Spike Sequence. For panels A and C, the Y axis represents codons, while the X axis represents positions along the Spike sequence for which entropy exceeds 0.1. Within the black boxes are codons synonymous to the WT codon, and within the red box is the WT codon at that position. For panels B and D, the Y axis represents nucleotides, while the X axis represents positions along the Spike sequence for which entropy exceeds 0.1 Within the red boxes are the WT nucleotides at that position. A) and B) show all SARS-CoV-2 Delta Spike protein sequences ranging from 10/2021-06/2022. C) and D) show all SARS-CoV-2 Omicron Spike sequences ranging from 11/2021-07/2021.

## Data Availability

The dataset(s) supporting the conclusions of this article is(are) available in the GISAID SARS-CoV-2 repository, [GISAID—gisaid.org]. The dataset(s) supporting the conclusions of this article is(are) included within the article (and its additional file(s)).

## Abbreviations

CAI: Codon adaptation index
dN/dS: Nonsynonymous/synonymous mutation ratio
WHO: World Health Organization
VOC: Variant of concern
CUB: Codon usage bias
E: Envelope gene
M: Membrane gene
N: Nucleocapsid gene
RSCU: Relative synonymous codon usage
GRY: Alpha clade
GH: Beta/Mu clade
GR: Gamma/Lambda clade
GK: Delta clade
GRA: Omicron clade
HA: Hemagglutinin
AT: Adenine + Thymine nucleotides
GC: Guanine + Cytosine nucleotides
AG: Purines
TC: Pyrimidines
