# Supplementary figures and images for: Analysis of 3.5 million SARS-CoV-2 sequences reveals unique mutational trends with consistent nucleotide and codon frequencies
Source: Virol J. 2023 Feb 17;20:31. doi: 10.1186/s12985-023-01982-8 (PMC9936480; doi:10.1186/s12985-023-01982-8)

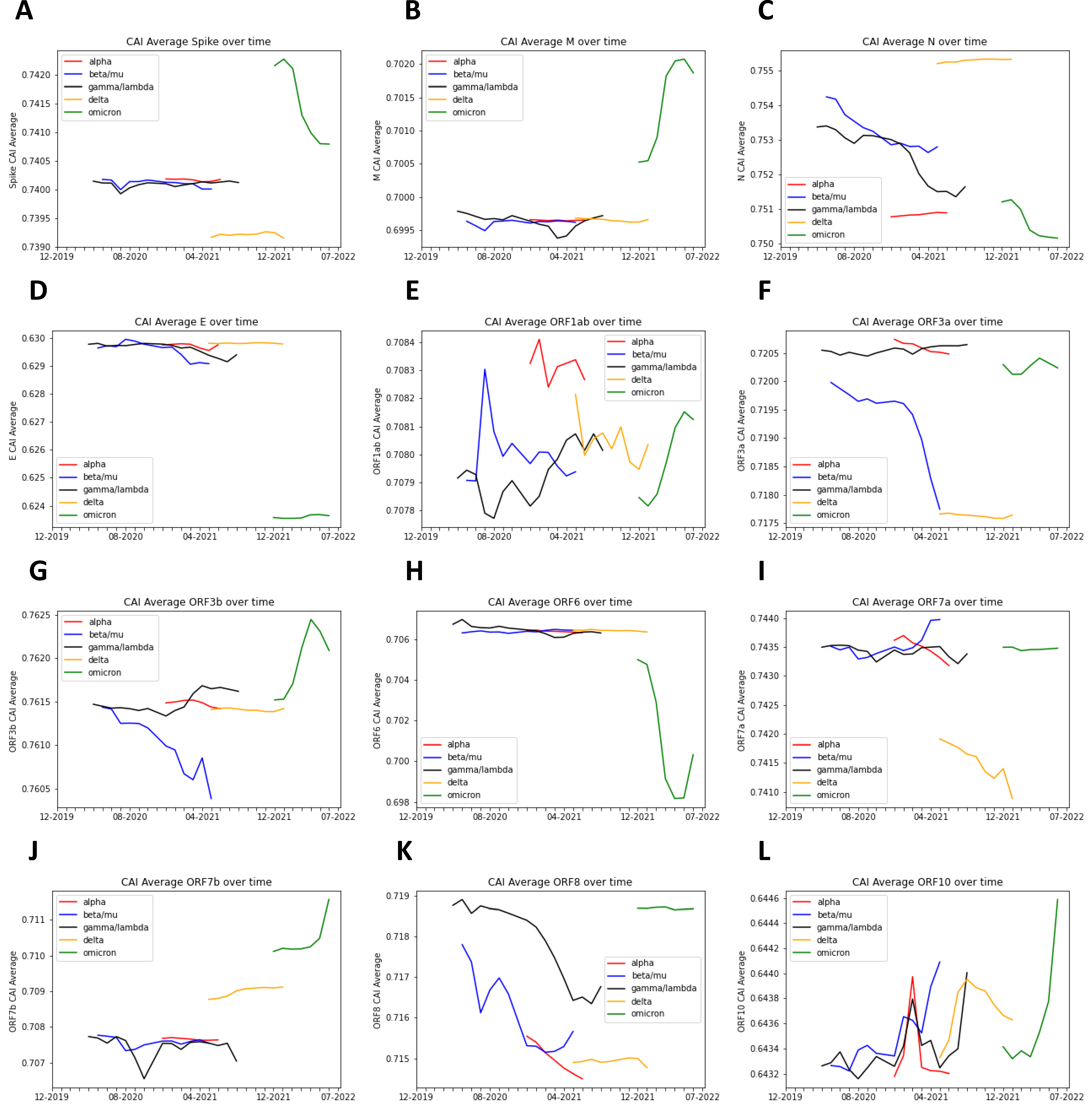

Supplement: Supplementary file 25 — Additional file 25. Figure S13: Line Plots Showing Average CAI Over Time for All Genes. Panels A-L refer to Spike, M, N, E, ORF1ab, ORF3a, ORF3b, ORF6, ORF7a, ORF7b, ORF8, and ORF10, respectively. Data is plotted only for the dates that are bolded & italicized in Table 1. [file 12985_2023_1982_MOESM25_ESM.png]

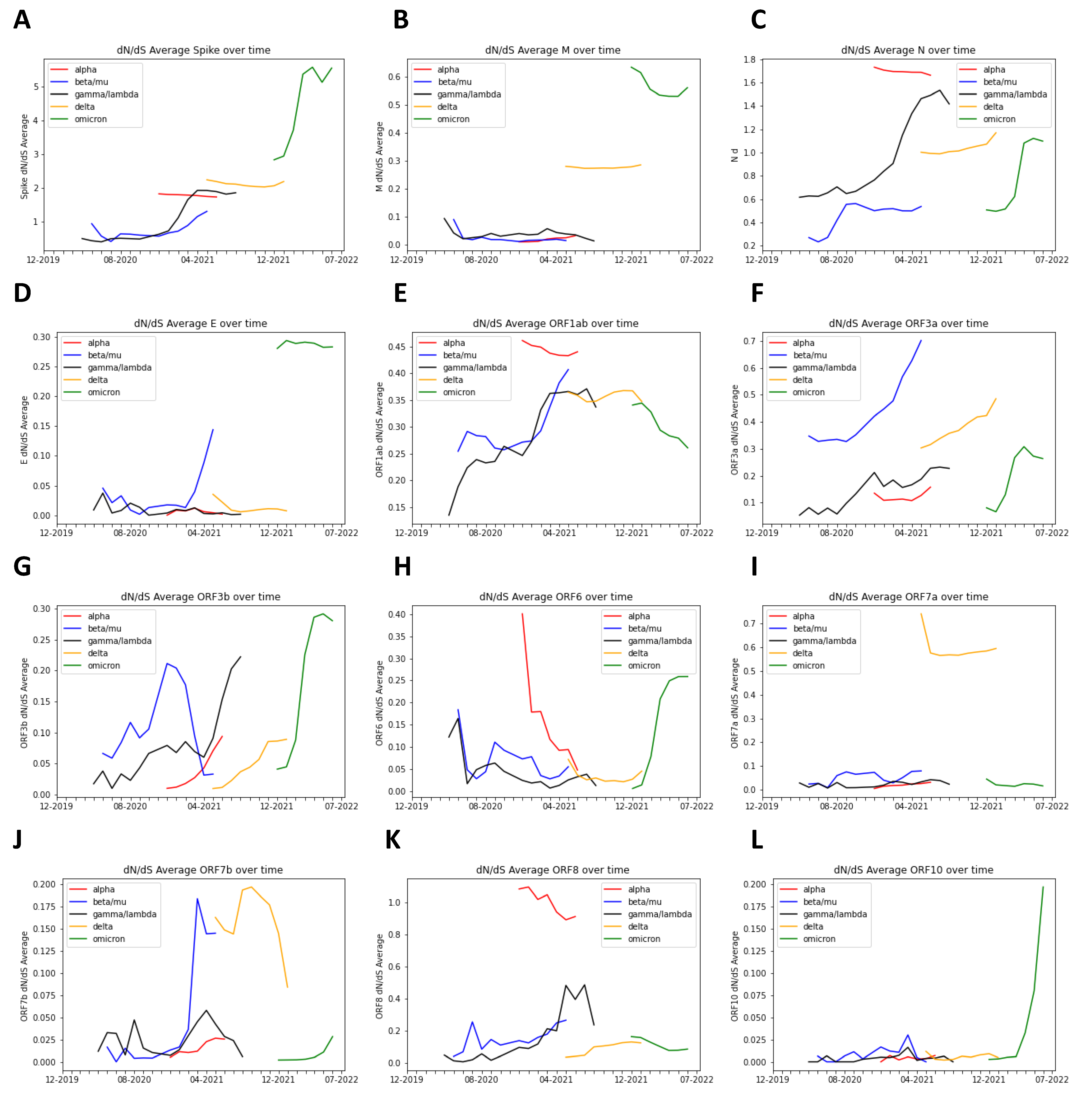

Supplement: Supplementary file 26 — Additional file 26. Figure S14: Line Plots Showing dN/dS Ratio Over Time for All Genes. Panels A-L refer to Spike, M, N, E, ORF1ab, ORF3a, ORF3b, ORF6, ORF7a, ORF7b, ORF8, and ORF10, respectively. Data is plotted only for the dates that are bolded and italicized in Table 1. [file 12985_2023_1982_MOESM26_ESM.png]

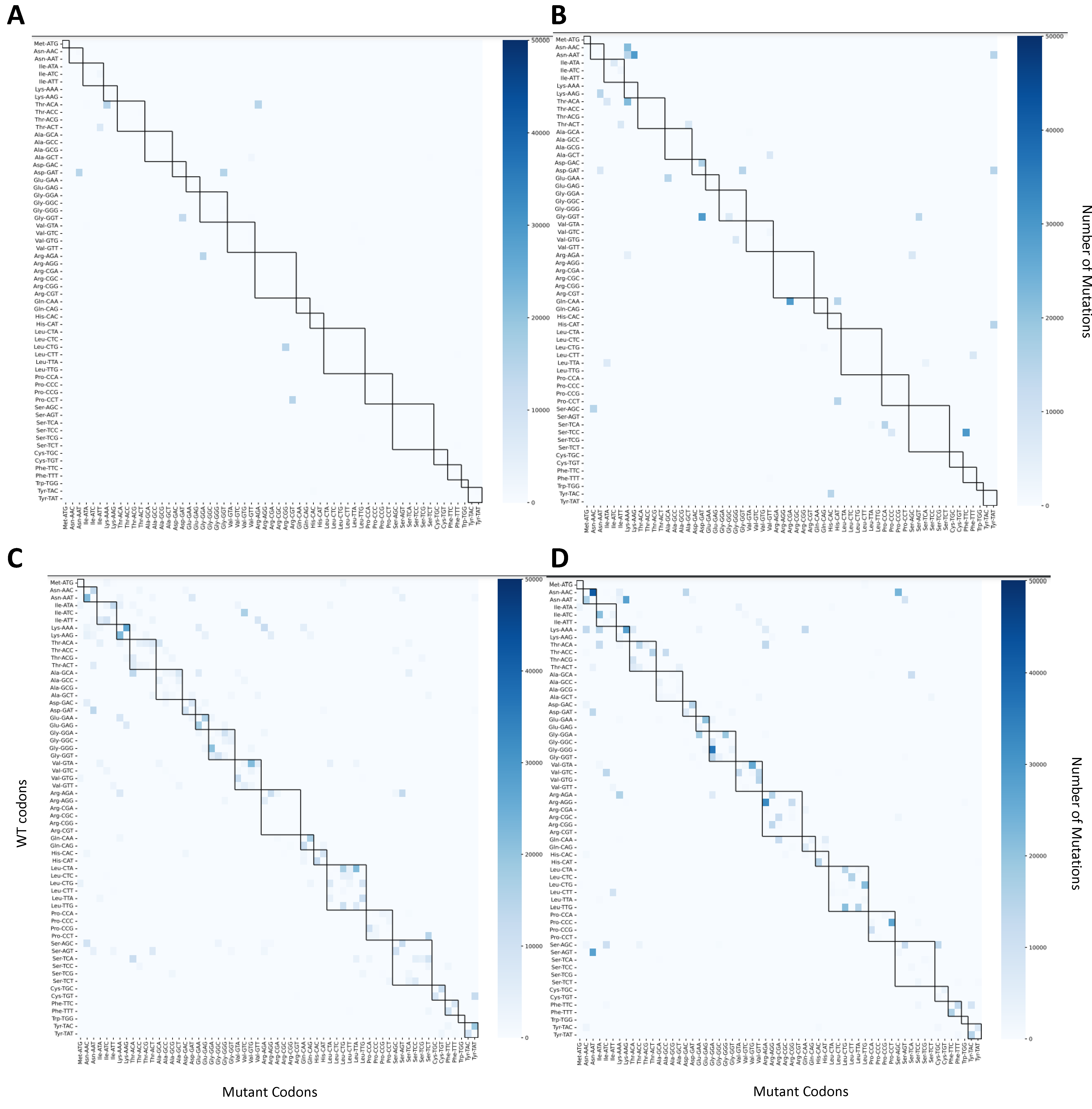

Supplement: Supplementary file 27 — Additional file 27. Figure S15: Missense and Synonymous Mutations for SARS-CoV-2 Spike and Influenza Virus HA, Normalized for Sequence Count. Both axes represent individual codons, with each point on the heatmap representing a mutation from one codon to the other. Darker points on the heatmap represent more frequent mutations. Mutations within the black boxes are synonymous, and mutations outside these boxes are nonsynonymous. Each panel shows a heatmap generated from 15,000 sequences of A) SARS-CoV-2 Delta Spike ranging from 10/2020-06/2022, B) 15,000 SARS-CoV-2 Omicron Spike ranging from 11/2021-07/2022, C) 15,000 H1N1 HA ranging from 01/1989-02/2019, and D) H3N2 HA ranging from 12/1980-06/2022. [file 12985_2023_1982_MOESM27_ESM.png]

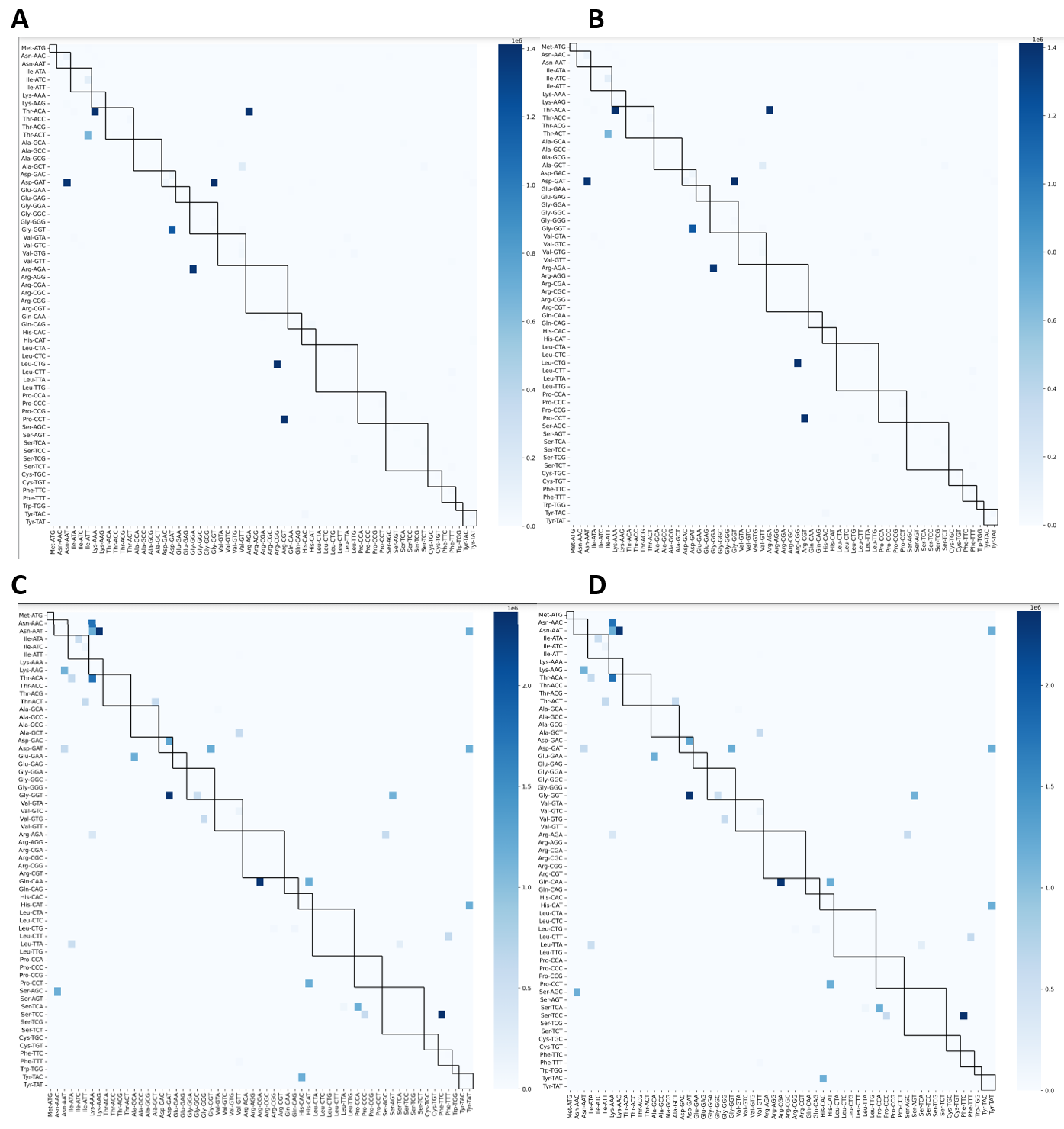

Supplement: Supplementary file 28 — Additional file 28. Figure S16: Missense and Synonymous Mutations for SARS-CoV-2 Spike, Omicron and Delta, Comparison Between Full Cohort of Mutations and Rare Mutations Excluded. Both axes represent individual codons, with each point on the heatmap representing a mutation from one codon to the other. Darker points on the heatmap represent more frequent mutations. Mutations within the black boxes are synonymous, and mutations outside these boxes are nonsynonymous. Individual panels show heatmaps representing A) 1,407,663 SARS-CoV-2 Delta Spike Protein Sequences ranging from 10/2020-06/2022, B) 1,407,663 SARS-CoV-2 Delta Spike Protein Sequences ranging from 10/2020-06/2022, with mutations that are presenting in 2 or fewer sequences excluded, C) 1,195,200 SARS-CoV-2 Omicron Spike protein sequences ranging from 11/2021-07/2022, and D) 1,195,200 SARS-CoV-2 Omicron Spike protein sequences ranging from 11/2021-07/2022, with mutations that are presenting in 2 or fewer sequences excluded. [file 12985_2023_1982_MOESM28_ESM.png]

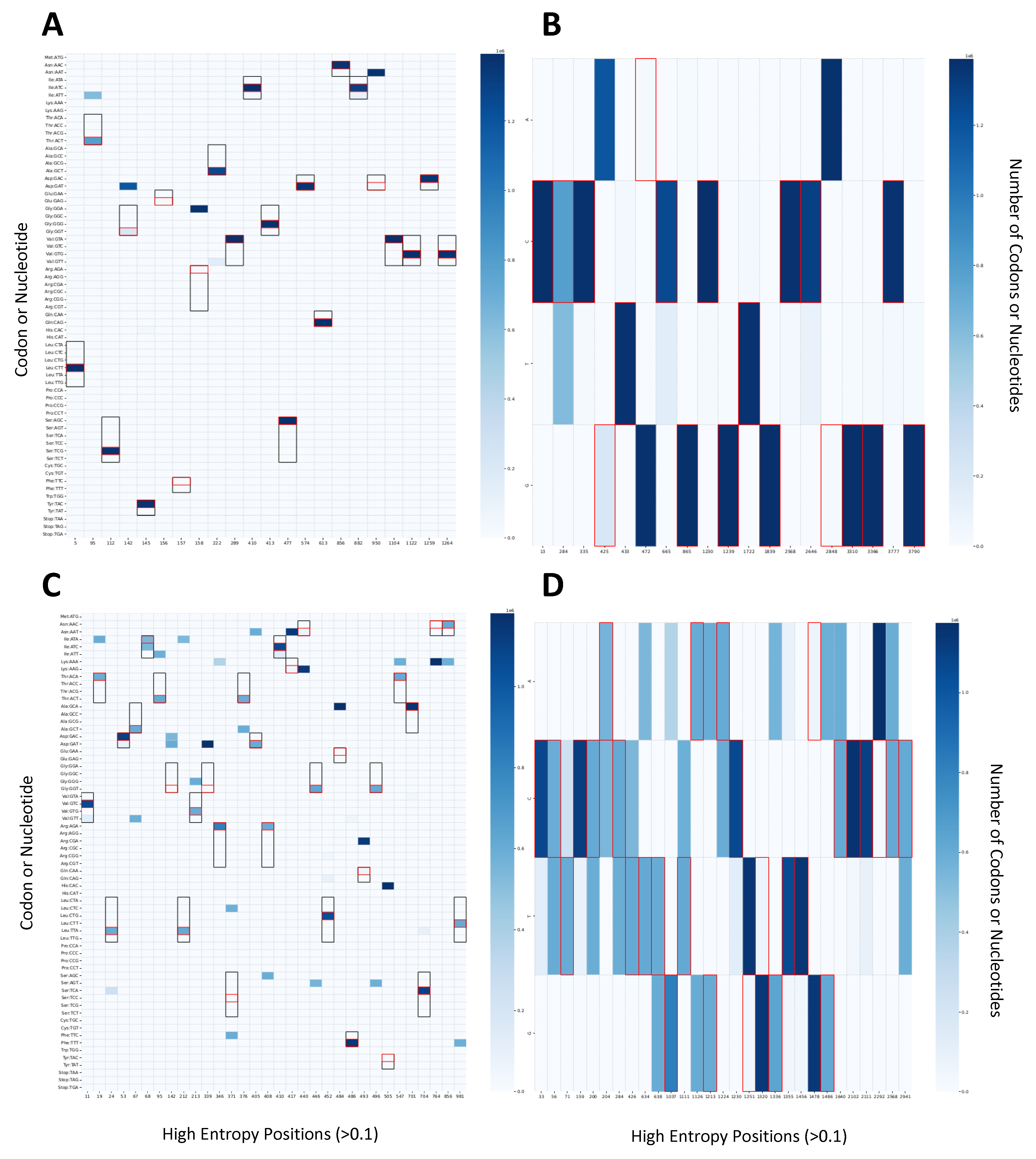

Supplement: Supplementary file 29 — Additional file 29. Figure S17: Missense and Synonymous Mutations at High Entropy Positions Along Spike Sequence. For panels A and C, the Y axis represents codons, while the X axis represents positions along the Spike sequence for which entropy exceeds 0.1. Within the black boxes are codons synonymous to the WT codon, and within the red box is the WT codon at that position. For panels B and D, the Y axis represents nucleotides, while the X axis represents positions along the Spike sequence for which entropy exceeds 0.1 Within the red boxes are the WT nucleotides at that position. A) and B) show all SARS-CoV-2 Delta Spike protein sequences ranging from 10/2021-06/2022. C) and D) show all SARS-CoV-2 Omicron Spike sequences ranging from 11/2021-07/2021. [file 12985_2023_1982_MOESM29_ESM.png]
